# What Twitter teaches us about patient-provider communication on pain

**DOI:** 10.1371/journal.pone.0226321

**Published:** 2019-12-26

**Authors:** Yasmin M. Kloth, Kenneth M. Deutsch, Katy A. Danielson, Julie Strack, Catherine Law

**Affiliations:** 1 *All of Us* Research Program, National Institutes of Health (NIH), Bethesda, MD, United States of America; 2 JPA Health Communications, Boston, MA, United States of America; 3 National Center for Complementary and Integrative Health (NCCIH), National Institutes of Health (NIH), Bethesda, MD, United States of America; University of Zurich, SWITZERLAND

## Abstract

The objective of this study was to understand how pain patients and health care providers (HCPs) are engaging on Twitter and what insights this engagement might provide. By identifying how information is spread by and between these audiences, organizations such as patient advocacy groups may be better poised to develop and share materials that facilitate online communication between HCPs and pain patients, with an end goal of improving a shared decision-making process around pain management. We analyzed the Twitter audiences most engaged on pain topics by conducting a Social Network Analysis (SNA) of a large network of connected users on Twitter. The analysis segmented users based on the sources they cited and measured their influence based on who follows them. As a point of comparison, we also conducted an SNA of Twitter audiences most engaged on oncology topics. Oncology was chosen as a comparison due to what was perceived to be a highly developed online network of both patients and physicians. The populations included in this research included 12,086 accounts that were highly engaged on pain-related topics, and 12,617 accounts that were highly engaged on oncology-related topics. Network statistics were generated for variables including: word use, sources cited, retweets, and mentions. We also statistically analyzed the Twitter follow relationships among select HCPs and patient groups within each SNA. The creation of separate pain and oncology SNAs allowed the team to compare relationships and engagement related to these topics. We found that on Twitter, pain patients and providers appear to interact less than oncology patients and providers. Pain patients do not appear to follow medical professionals or share medical or health-related information on Twitter to the same extent as oncology patients. In addition, we found that pain patients do not communicate on Twitter in the same language as HCPs. Our results are important because they underscore that challenges in communication are not just problematic in face-to-face interactions, but also in digital social network (Twitter) interactions, serving as an additional roadblock to what can be shared decision-making opportunities around pain management. Contributing to this roadblock is access to quality information and a potential need for an online, evidence-based resource hub that could benefit the pain patient community in the same way that cancer.gov serves as a source of aggregated materials for oncology patients and HCPs. This study is an illustration of how social media networks like Twitter can be used to better understand the relationships, language gaps, and shared resources between pain patients and providers and offers a template for using digital social network (Twitter) interactions to research other difficult-to-treat or rare disease states.

## Introduction

Pain is the most common reason for seeking medical care, and approximately 25 million American adults suffer from daily chronic pain [1]. With Americans struggling to treat their chronic pain, the use of opioids for pain management has come under scrutiny due to a rise since 1999 in use and serious complications such as addiction and overdose deaths [2]. As reported by the National Pain Strategy (NPS), “prescribing practices, marketing, and misleading information on safety drove a steady and significant increase in the number of opioid prescriptions dispensed, rising from 76 million in 1999 to 219 million in 2011 [3]”.

To improve health outcomes among pain patients, there is a need to understand how patients and health care practitioners (HCPs) communicate about pain management or treatment plans [4]. Due to the physical and emotional complexities of pain, patients often struggle to articulate the level of distress they face, and HCPs are often unequipped with the time or the tools needed to provide these patients with a supportive response or well-rounded and integrated treatment plans [5, 6]. The National Pain Strategy (NPS), the federal government’s first coordinated plan for reducing the burden of chronic pain, emphasizes the need for strong communication between HCPs and patients. The NPS states that “Improvements in professional education about state-of-the-art care for pain, in all its dimensions, including better communication, empathy, cultural sensitivity, and risk management will yield significant care improvements [7]”.

Research has found that a shared decision-making approach to pain management—an approach that involves engaged collaboration between patients and HCPs—is an important part of an overall pain treatment plan [8]. But there are hurdles to this type of approach, including restraints on time for both providers and patients [9]. Trust between pain patients and providers, although not very well understood, also plays a key role in how patients and providers communicate and may have an effect on a patient’s health outcomes [10].

To add complexity to these patient‒provider conversations, health discussions are not just taking place in physicians’ offices. Digital platforms, such as websites and social media sites (e.g., Facebook and Twitter), have become places where people not only find health information, but where they congregate, connect, and discuss their health questions and concerns with others who may be experiencing similar circumstances [11]. “One-in-four (26%) adult internet users say they have read or watched someone else’s health experience about health or medical issues in the past 12 months. And 16% of adult internet users in the U.S. have gone online in the past 12 months to find others who share the same health concerns,” according to 2014 Pew Research Center statistics [12].

The trend continues with the next generation. Not only are 9 out of 10 teens and young adults going online for health information, “about four in ten (39%) say they have gone online to try to find people with health conditions similar to their own, using methods such as participating in online forums or closed social media groups on specific issues, doing hashtag searches on social media, or following people with similar health conditions,” according to a 2018 report from Hopelab and Well Being Trust [13].

In addition, learning networks (both digital and in-person) have been used in health care settings to connect knowledgeable communities—patients, providers, and researchers—to work on solutions for difficult-to-treat conditions [14]. Research has shown that learning networks improve health outcomes as participants from different areas of health care work toward a common goal [15]. It is in this environment that the internet becomes a powerful and empowering tool for patients to look for health information [16] and to make online connections with other like-minded individuals and communities.

As a result, as people turn toward the internet for their health information, for community, and for connection, it’s important to study their online behaviors—how and where people get their information—in an effort to improve how that information is delivered and consumed. The need for quality, evidence-based resources is not going unseen. A call to assess patient education materials and decision support resources as key components for effective pain care were reflected in the 2018 Federal Pain Research Strategy issued by the National Institutes of Health [17].

This paper seeks to closely examine how pain patients and providers communicate on Twitter and to explore how these conversations might help inform the information needs of pain patients.

### Why Twitter?

Twitter is an online news and social networking platform where users post and interact using messages called “tweets.” While it is not the most used social network, it has the research advantage of consisting of conversations and follow relationships that are almost entirely in the public domain. Twitter’s use by health professionals is documented [18] and though not every person uses the platform, there are people commenting about a wide variety of topics. With this broad and varied range of topics comes tremendous research opportunity. For example, recent research explored the use of Twitter to communicate about and congregate around 379 different health conditions, but the researchers also found limitations in previous research [19]. Namely, these types of studies “have typically focused on a single, or a very limited set of related health conditions, usually different kinds of cancers. Very little work has compared Twitter communities of different health conditions which could uncover potential trends and highlight popular health topics [20].” This allows for opportunities in research to better understand the conversations and comparisons around different disease states and health conditions.

Twitter users create their own lenses to view the rest of the platform. By choosing users to follow, they are creating blinders with which they only view content based on the interests of the people they follow. This behavior allows for analysis on how health communicators can effectively share information with specific audiences or view the ways information flows. Twitter also has an advantage in that users tend to cite other publications in their tweets, such as via linking a URL or mentioning the publication’s account.

Despite limited character allowances in tweets, the ability to add citations brings in large amounts of meaningful data. In fact, recent research shows that Twitter citations of medical research accurately predicts the citations of those publications in future published articles [21, 22].

### Study objective

In face-to-face interactions between patients and HCPs, research has found that differences in points of view [23], as well as differences in language, create a disconnect in communication between pain patients and providers, and may contribute to the challenges of pain management and treatment [24]. Because of this disconnect and a need to improve our understanding of how pain communities operate on Twitter, we hypothesized that pain patients and HCPs, when compared with oncology patients and providers, engage with different audiences and share information from different sources on Twitter, indicative of a communications gap that exists online between pain patients and HCPs. To address this hypothesis, this paper will:

1. Define the differences and similarities in Twitter relationships and identify what the similarities and differences in engagement among patients and HCPs within a sample of social media accounts tell us.
2. Compare word use and citations among pain patients and HCPs. This evaluation focused on comparing the types of information sources that these audiences are sharing (medically focused sources, general consumer sources, or other types of sources). This analysis allowed us to evaluate whether both groups are sharing medically focused sources, or whether one or both groups may be sharing more general or consumer-focused sources of information. As part of this analysis, we also evaluated the extent to which both audiences are discussing pain-related topics, to confirm that a high amount of their respective citations were likely shared in relation to pain-focused topics.
3. Outline the challenges and limitations in addressing any differences between the audiences evaluated.

## Materials and methods

Our research aims to define the audiences with whom patients and HCPs engage online and the information they share about pain management, specifically on Twitter. To define the audiences, we worked with Graphika Inc. to analyze the Twitter audiences most engaged in pain and oncology topics by conducting two separate Social Network Analysis (SNA) maps focused on these respective topics. We assessed the structure of Twitter relationships within the Pain and Oncology SNAs and compared the strength of the relationships between the patient and HCPs audiences within each SNA. This analysis was primarily focused on evaluating whether any potential gaps in communication exist between the patient and HCP audiences in the Pain SNA, when compared with the Oncology SNA. The creation of two separate SNAs provided comparisons between the audiences engaged on these issues, allowing us to define trends related to the communication patterns of the audiences within each map. While the main focus of the research was on the Pain SNA, oncology was chosen as a comparison due to what was perceived to be a highly developed online network of both patients and physicians.

An SNA is the analysis and visualization of large networks of connected users on Twitter, and it provides insight into the social engagement of accounts focused on the specific topics and flow of information between these accounts. Network analysis has been used frequently in past research to study health-related issues and is an “approach to research that is uniquely suited to describing, exploring, and understanding structural and relational aspects of health [25].” SNAs of Twitter, specifically, have been used in past health research to evaluate “the relationship and interactions between Twitter users about a certain topic,” such as a study that assessed “the connections between patients with cancer on Twitter and sought to identify the hubs, or Twitter users with the greatest connectedness to other users within the network [26].” SNA maps are comprised of individual Twitter accounts, which are connected to other accounts in the map via social relationships. The initial mapping processes, which were conducted on July 27, 2017, for the Pain SNA and May 19, 2017 for the Oncology SNA, were based on follow relationships and then further segmented by audiences using attentive clustering (grouping users based on the specific sources that they cite).

To begin the creation of the SNA maps, we generated “seed lists” by compiling a sample of accounts with a focus on topics related to pain and oncology. The pain seed list (12 users) was smaller than the oncology seed list (22 users) as there were fewer accounts that were focused just on the subject matter. Starting with this list, all their followers, the users they followed, and individual tweets were collected. Collection also included new accounts discovered during the mining process.

The Twitter dataset was collected using Twitter’s Streaming Application Program Interface (API). To determine the placement of users into clusters, Graphika’s algorithm was utilized. This algorithm, which has been used in previous research focused on social network analysis, also generated network statistics, including measurements of influence based on follow relationships within the map [27].

A Fruchterman-Reingold visualization algorithm was used in both maps to represent the patterns of connection between these accounts. It arranges the accounts in a visualization through a centrifugal force that pushes nodes to the edge and a cohesive force that pulls strongly connected nodes together.

This method of segmenting users and generating broad observations about associations is an iterative process drawing on qualitative, quantitative, and computational methods. To create a map of segments and groups, we used a bipartite graph to provide a structural similarity metric between the accounts within the map, which is used in combination with a clustering algorithm to segment the map into distinct communities. Hierarchical agglomerative clustering was used to automatically generate segments and groups from sampled data.

The identification of which audiences (patients, HCPs, etc.) each cluster was composed of was determined through reviewing the following: the members of the clusters (self-reported Twitter data); the sources they cited; the users they mentioned; and the users they followed. The review of the clusters was completed by prioritizing the users within each cluster who had the largest number of followers within the map.

The outcome of this process was the creation of two separate SNA maps, focused on pain and oncology. The top accounts—12,086 accounts in the Pain SNA and 12,617 accounts in the Oncology SNA, as determined by those most well-connected to others in the SNA—were included in this research. Network statistics were generated for variables, including word use, sources cited, retweets, and mentions. The sources that each of the audiences cited (Tables 1 and 2) were collected and tabulated between July 17, 2017, and March 31, 2018.

**Table 1:** Ranked word pairs cited by pain patients and HCPs.

	Chronic Pain Patients	HCPs	Pain Medicine & Research	Nonpharmacologic Pain Treatment
chronic pain	1	21	1	2
health care	2	1	3	8
feel like	3	31	33	29
chronic illness	4	46	29	55
looks like	5	14	7	18
mental health	6	2	4	19
look like	7	29	27	28
long term	8	3	5	12
sounds like	9	38	26	35
feel better	10	45	50	31
study finds	17	5	22	27
health insurance	18	8	56	52
young people	19	34	8	33
pain relief	21	44	9	24
study shows	26	10	15	36
pain management	28	36	2	14
senate health	29	6	57	38
public health	34	7	16	26
opioid epidemic	37	9	12	39
low pain	39	47	6	1
physical activity	40	33	19	5
primary care	41	4	13	21
physical therapy	43	41	48	3
evidence based	47	18	10	10
neck pain	48	53	55	7
systematic review	53	39	25	9
persistent pain	54	57	21	6
manual therapy	59	58	59	4

Data collected from June 27 until July 27, 2017.

Numbers indicate the order of word pairs mentioned; the top 10 for each audience was included along with the actual rank for the other audiences. A 1 ranking is the most frequently mentioned word pair for that audience.

**Table 2:** Sources cited by pain patients and provider audiences.

		Provider Audiences		
	Chronic Pain Patients	HCPs	Pain Medicine & Research	Nonpharmacologic Pain Treatment
nytimes.com	1	1	3	1
bbc.co.uk	2	5	1	4
bestbuy.com	3	4715	3784	2948
theguardian.com	4	3	2	2
amazon.com	5	126	683	262
cnn.com	6	3	15	73
washingtonpost.com	7	4	21	34
thehill.com	8	6	28	25
pizzazzbookpromotions.wordpress.com	9	4716	3786	2951
ibotta.com	10	8928	3788	2956
politico.com	13	10	76	88
wsj.com	17	5	30	32
independent.co.uk	25	68	4	35
bbc.com	36	28	9	95
npr.org	57	9	84	29
statnews.com	**76**	**2**	**10**	**62**
khn.org	**198**	**7**	**132**	**436**
washingtonpost.com	265	75	37	3
theguardian.com	328	94	27	8
ft.com	373	79	8	190
thehill.com	428	80	34	5
wired.com	461	123	97	7
who.int	**527**	**36**	**5**	**124**
politico.com	569	90	96	6
jamanetwork.com	933	8	20	57
huffingtonpost.com	1099	96	123	10
nhs.uk	**1250**	**3020**	**7**	**380**
bloomberg.com	1414	153	83	9
bmj.com	**1502**	**54**	**6**	**64**

Data collected between July 2017 and March 2018.

Numbers indicate the order of URLs cited; the top 10 for each audience was included along with the actual rank for the other audiences. A 1 ranking is the most frequently cited URL for that audience.

We also analyzed the top 12,000 pairs of words within each audience in the Pain SNA, using data collected from June 27 to July 27, 2017. This analysis allowed us to confirm that the Pain SNA captured measurable conversations focused on pain topics. It also allowed us to summarize the thousands of tweets that took place in the Pain SNA, with the goal of identifying the topics that pain and provider audiences are discussing with the most frequency.

### Defining the study populations

The figures represent the populations included in the study. The figures are visualizations of the nodes included in each SNA analysis conducted for this study and have been included as a visual illustration of the audiences evaluated within this study.

Within the visualizations, each dot represents a node, which is the Twitter account associated with a specific individual or organization within each SNA. Size and position of the nodes are based on their relative follow relationships within the network: the largest nodes have the most follow relationships within the map, and nodes are positioned near the accounts that they follow within the visualization. Color is determined by attentive clustering, based on the sources that the organizations and individuals cite within their posts. The clusters within the Pain and Oncology SNA maps included in this study are organized into overall audience groups, which are shown within the following figures.

**Fig 1:**
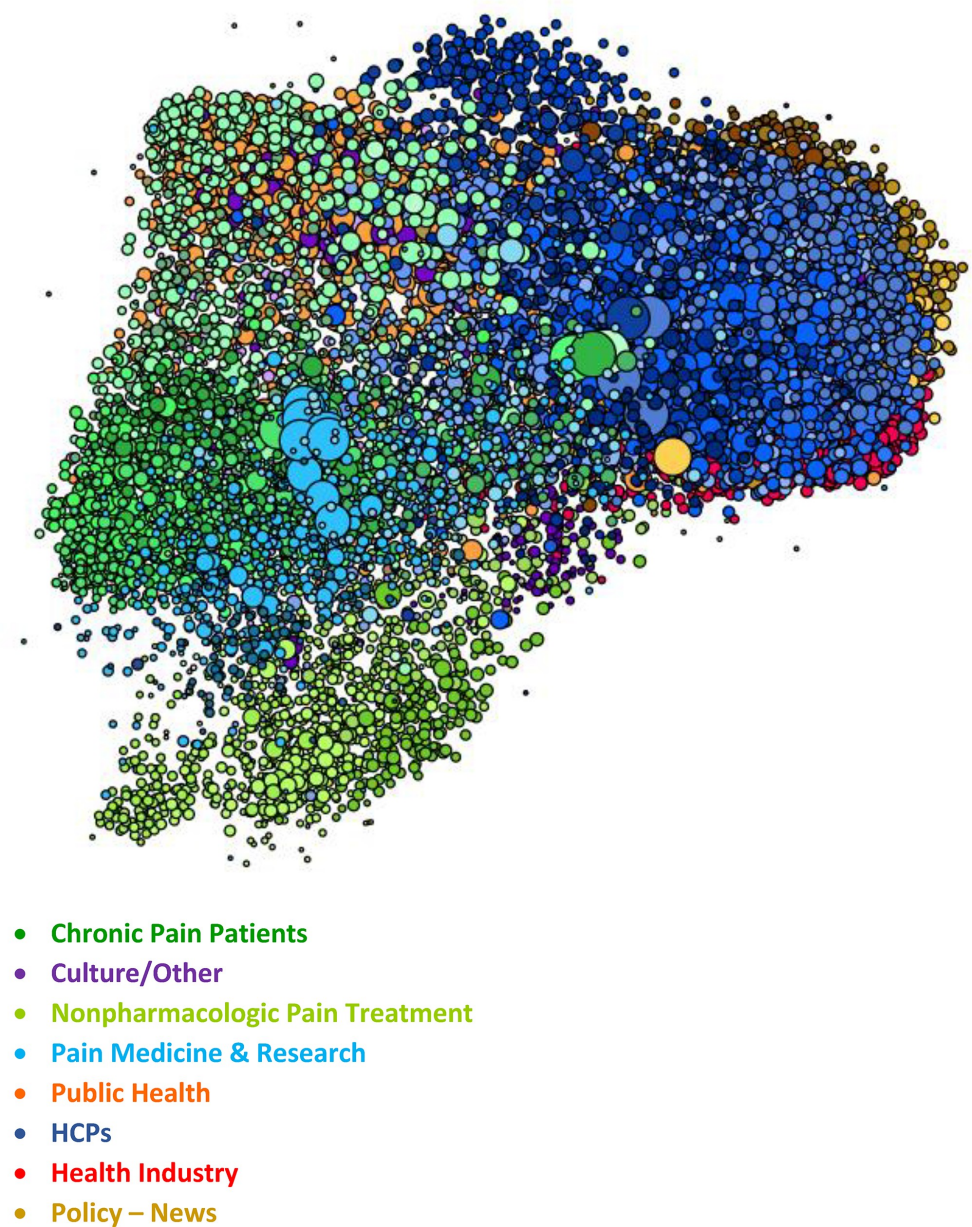
Pain SNA overview. Within the visualization, each dot represents a node. Size and position of the nodes are based on follower relationships within the network, and their color is based on attentive clustering. The figure shows the size, positioning, and color of the nodes within the primary audience groups that comprise the Pain SNA map.

The Pain SNA (Fig 1) is composed of 47 unique, attentive clusters organized into eight audience groups.

**Fig 2:**
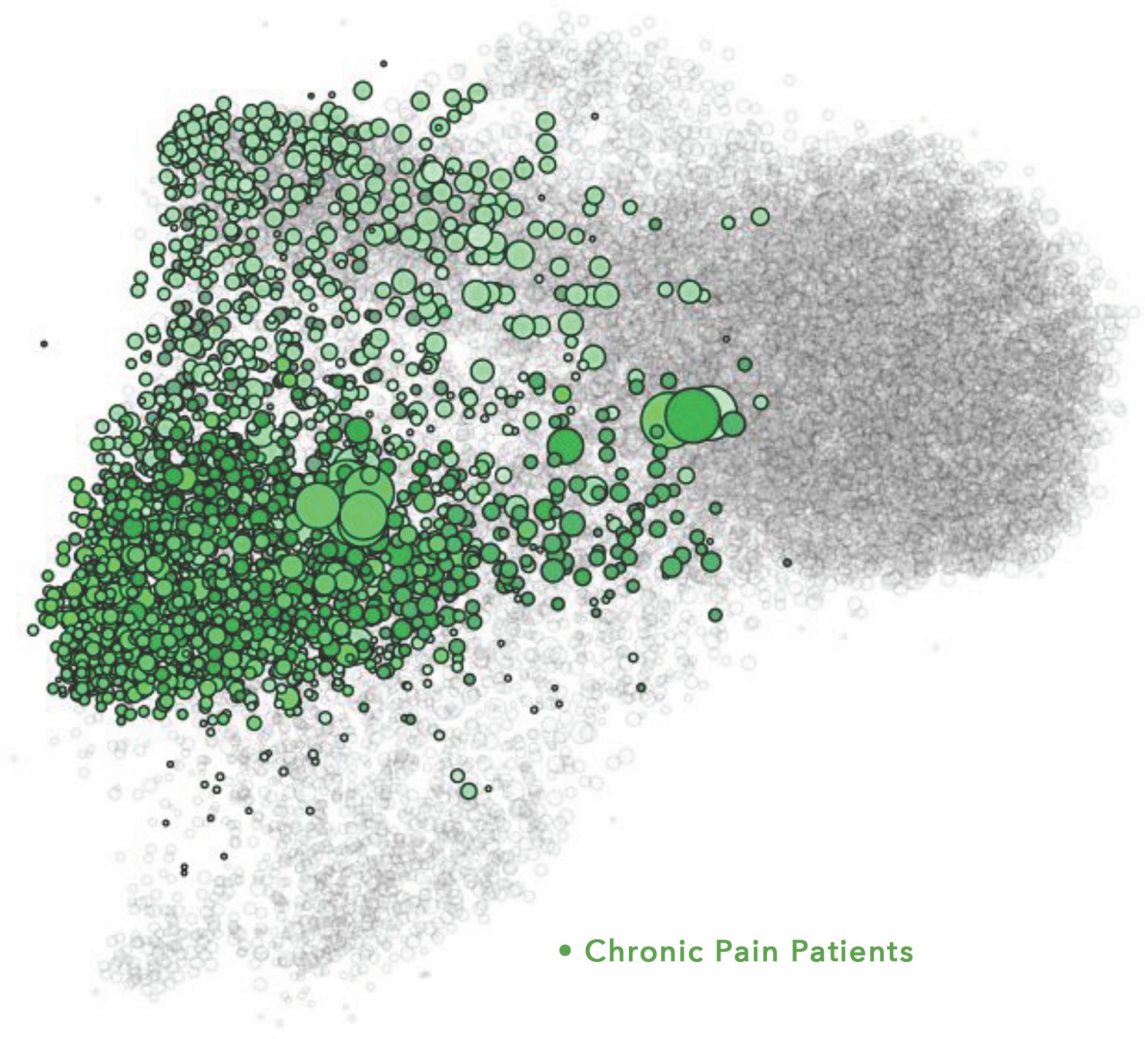
Chronic pain patients audience. Within the visualization, each dot represents a node. Size and position of the nodes are based on follower relationships within the network, and their color is based on attentive clustering. The figure shows the size, positioning, and color of the nodes that comprise the Chronic Pain Patients audience group within the Pain SNA map.

The Chronic Pain Patients (Fig 2) are individuals engaged with information on a variety of chronic pain conditions and treatment or management approaches. This audience was grouped into 10 separate clusters based on either the causes of pain or another common interest (Arthritis, Migraines, Neurological Patients, Chronic Conditions, RSD-CRPS [reflex sympathetic dystrophy/complex regional pain syndrome], Engaged Patients, Pain Patient Advocates, Pain Support–Women Centric, Patient Advocacy, and Spoonies). The Spoonies, a self-selected term for people living with a chronic disease, tended to cluster together based on the ways they developed to manage their energy, despite having many different types of pain among them.

**Fig 3:**
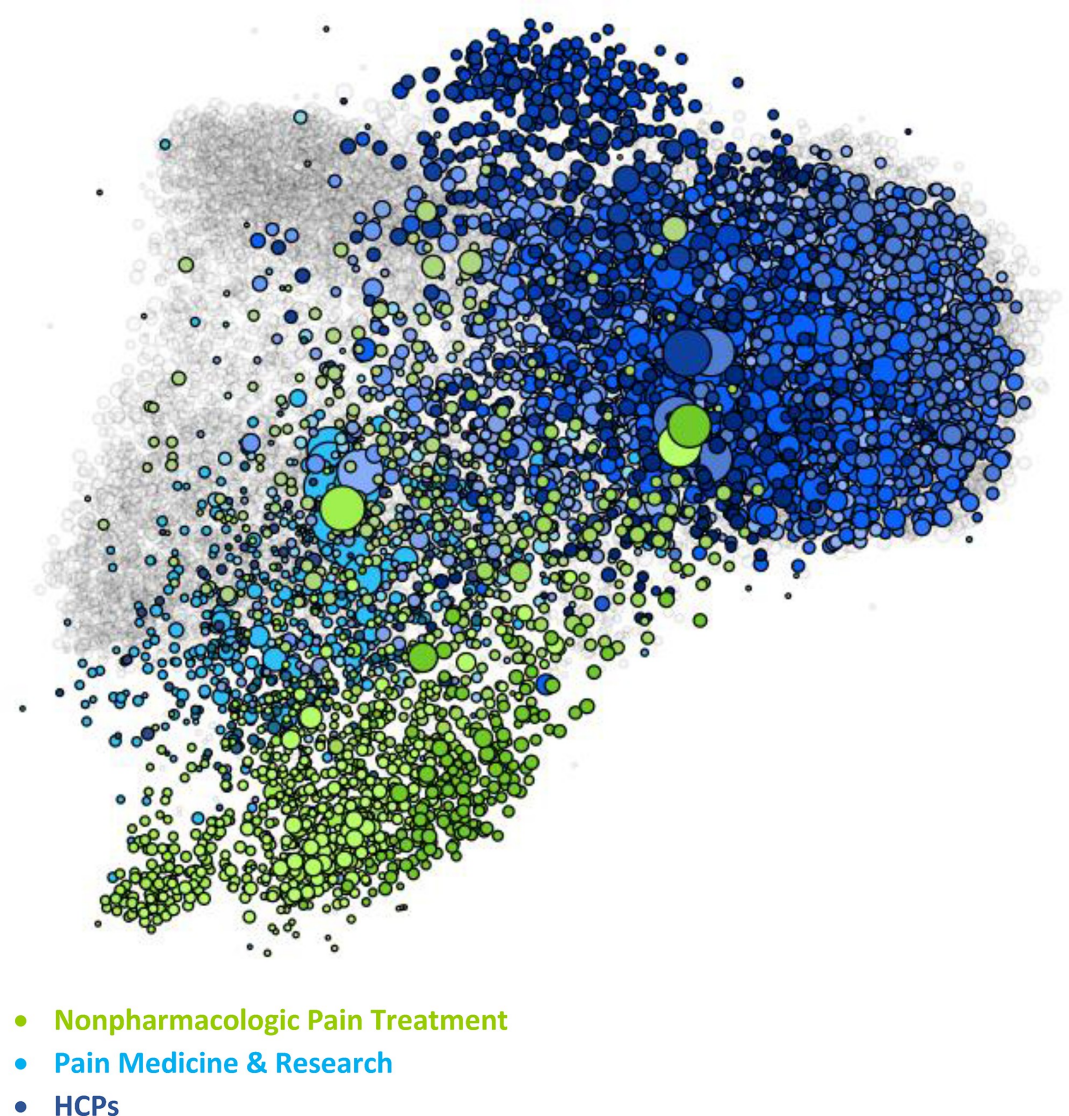
Pain health care provider audiences. Within the visualization, each dot represents a node. Size and position of the nodes are based on follower relationships within the network, and their color is based on attentive clustering. The figure shows the size, positioning, and color of the nodes that comprise the Nonpharmacologic Pain Treatment, Pain Medicine & Research, and HCPs audience groups within the Pain SNA map.

The Pain SNA includes three separate HCP audiences (Fig 3):

1. HCPs, who are general HCPs that deal with pain as well as many other medical issues.
2. Pain Medicine and Research, which is mostly composed of traditional HCPs who focus on either direct patient care or pain research.
3. Nonpharmacologic Pain Treatment, which includes traditional providers, such as physical therapists, and those who practice complementary health approaches, such as acupuncturists and massage therapists.

The other audiences (Culture/Other, Public Health, Health Industry and Policy–News) are all engaged on pain topics, but for the most part are not directly engaged with patient‒HCP communication. However, it should be noted that most of the Health Industry audience is composed of the pharmaceutical industry, which is highly engaged with the HCP audience.

**Fig 4:**
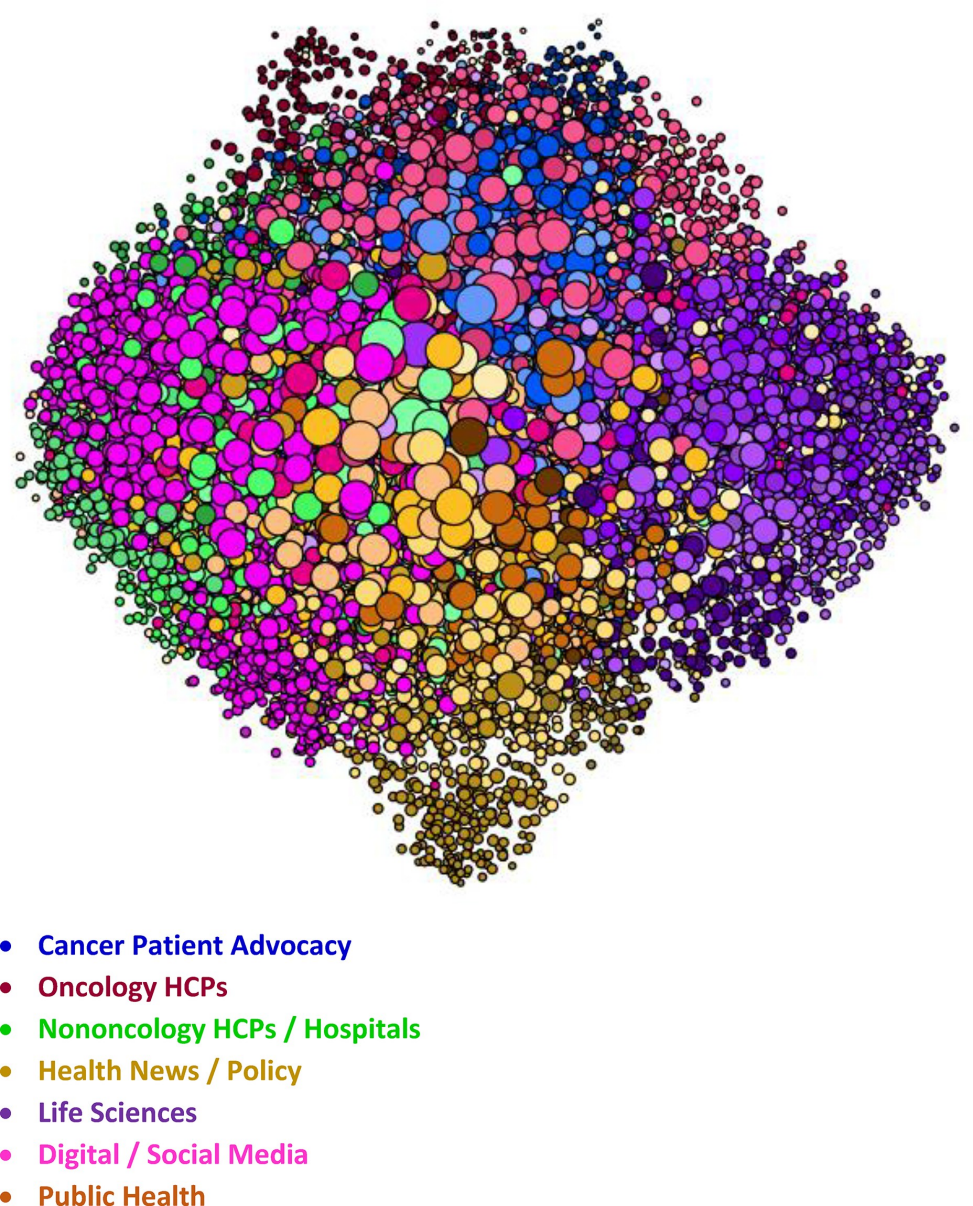
Oncology SNA overview. Within the visualization, each dot represents a node. Size and position of the nodes are based on follower relationships within the network, and their color is based on attentive clustering. The figure shows the size, positioning, and color of the nodes within the primary audience groups that comprise the Oncology SNA map.

The Oncology SNA (Fig 4) is comprised of 12,617 Twitter users in 48 attentive clusters, which are organized into seven audience groups. The patients are concentrated in the Cancer Patient Advocacy audience. As with the Pain SNA, the patients are clustered in disease focus (Melanoma, Pediatric, Gynecologic, Breast, and Lung) or by another common interest (Advocacy and Support).

**Fig 5:**
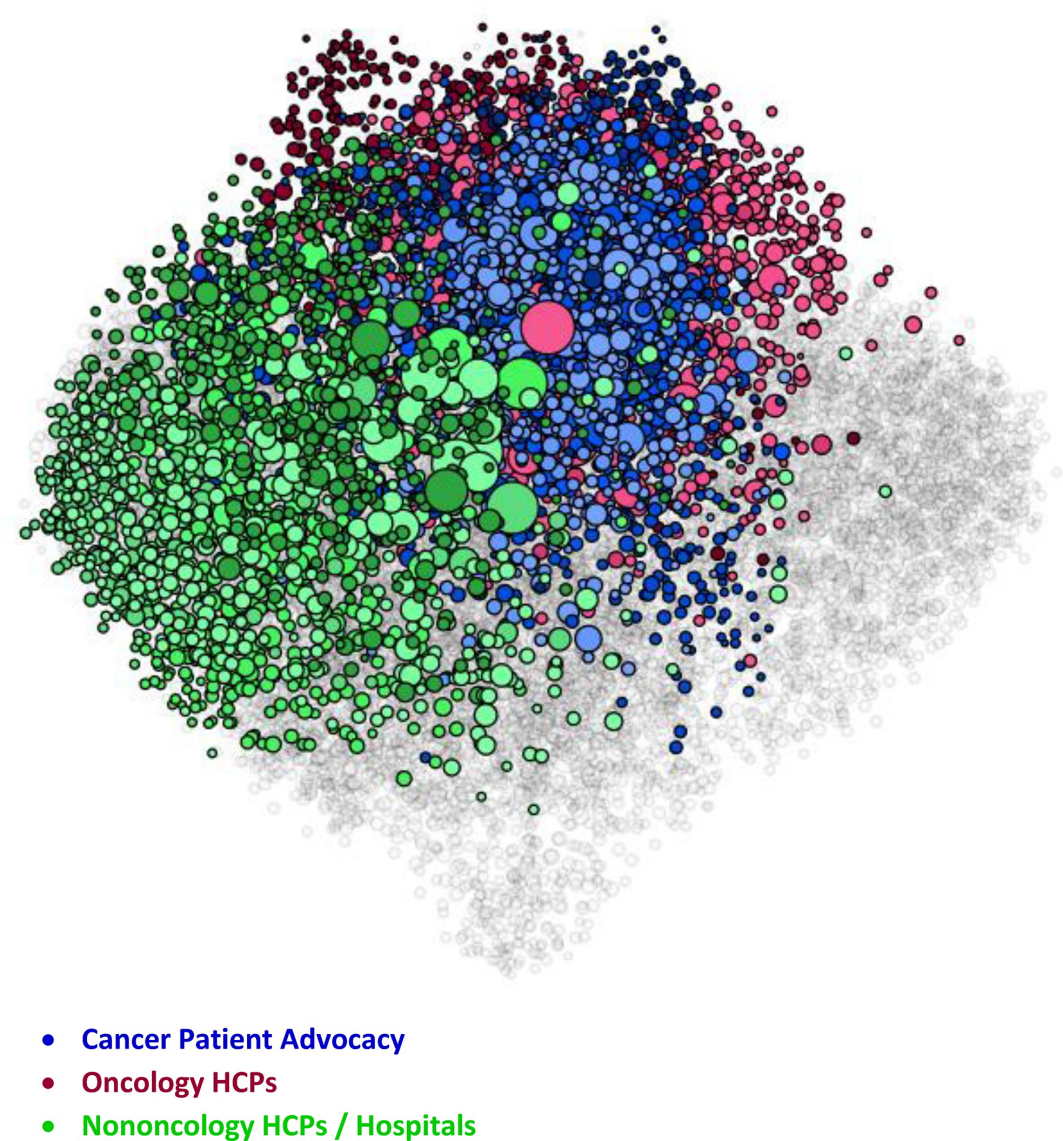
Oncology health care provider and patient audiences. Within the visualization, each dot represents a node. Size and position of the nodes are based on follower relationships within the network, and their color is based on attentive clustering. The figure shows the size, positioning, and color of the nodes that comprise the Cancer Patient Advocacy, Oncology HCPs, and Nononcology HCPs / Hospitals audience groups within the Oncology SNA map.

The HCPs (Fig 5) are concentrated into two audiences: Oncology HCPs (specialists) and Non-Oncology HCPs/Hospitals. The patient and the Oncology HCP audiences are tightly intertwined, while the other HCPs are not as engaged. There are several other audiences that are captured within the Oncology SNA.

### Analysis of follow relationships

The follow relationships between audiences were compared by collecting the total follows between groups, and then applying a density measure to correct for disparate audience sizes. The density measure normalized the data by weighting the total follows between each audience in accordance with its number of members. The normalization process assured that the size of a specific audience did not influence the findings on how audiences interacted, as the goal was to understand how members of an audience interacted with members of the other audiences.

**Fig 6:**
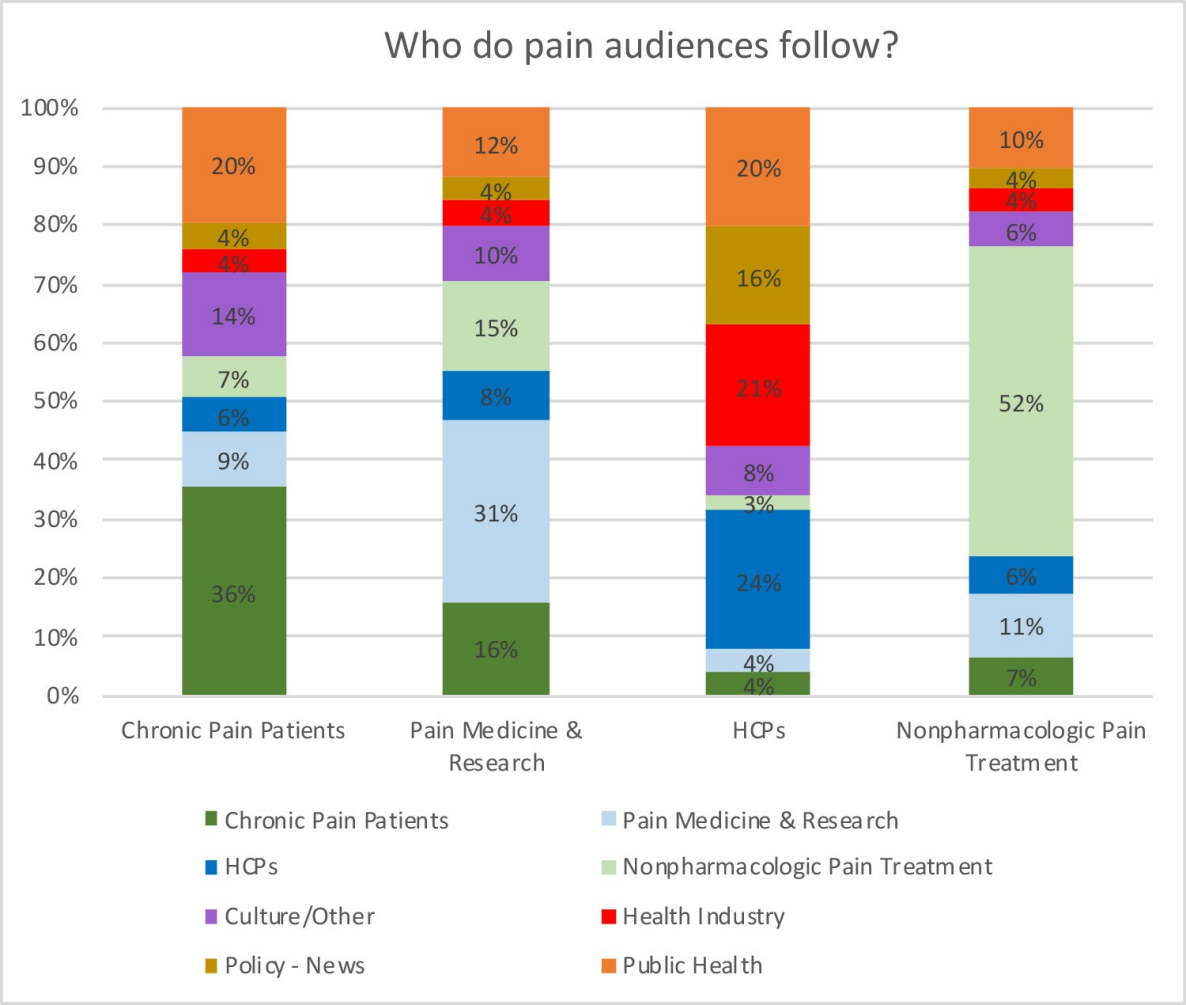
Who do pain audiences follow? The higher-than-average follow rates between oncology physicians and patients, and pain physicians and patients, were demonstrated by comparing each observed value to a random mixing baseline. The likelihood of finding the observed follow rates was calculated given a binomial distribution with the baseline probability. Statistical significance was determined at a 95 percent confidence level.

For example, within Fig 6, the total follows between the Chronic Pain Patients and Pain Medicine & Research audiences were normalized by applying the following equation: (total follows) / (members of Chronic Pain Patients audience * members of Pain Medicine & Research audience). The percentages included within the graphs were created with the normalized metrics (S1 File). (The total follows, before normalization, are included in the S1 File).

## Results

The following figures are visualizations of the comparative follow patterns between the select SNA audiences that are discussed within this section.

### 1. Define the structural differences and similarities in Twitter relationships

#### Comparative follower patterns—Comparative patient data

**Fig 7:**
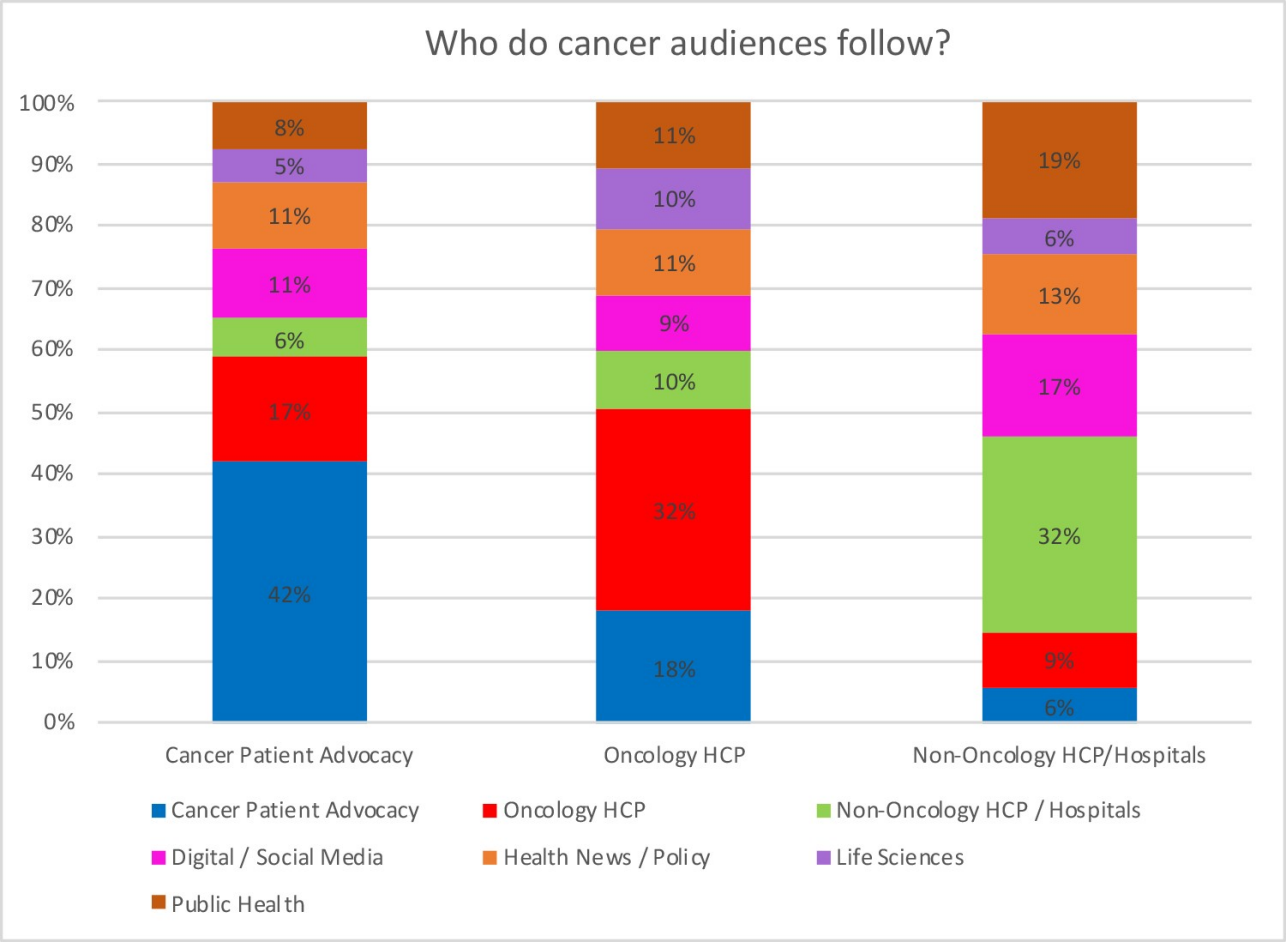
Who do cancer audiences follow?

Chronic pain patients follow other pain patients at a statistically higher rate than the amount, on average, that they follow all accounts within the Pain SNA map. Similarly, cancer patients are more likely than average to follow other cancer patients, when compared to a random mixing baseline (Fig 7). They also follow physicians who specialize in oncology at a statistically higher rate than the amount, on average, that they follow all accounts within the Oncology SNA map.

Chronic pain patients are less likely to follow other chronic pain patients than cancer patients are to follow other cancer patients (Fig 7). However, the follow patterns of chronic pain patients suggest they are less attentive to information shared by pain medicine and research specialists on social media than cancer patients are to cancer physicians (Oncology HCPs). Chronic pain patients are less likely to follow pain medicine and research specialists (9%) than cancer patients are to follow cancer specialists (17%).

Chronic pain patients and cancer patients are similarly likely to follow general HCPs (Figs 6 and 7). Both chronic pain patients and cancer patients follow other patients at a higher rate than the amount, on average, that they follow all accounts within their respective SNA maps. However, both patient audiences appear to follow general HCPs within their respective focus areas less than they follow specialist HCPs within their areas.

Pain and cancer specialists show similar follow patterns (Figs 6 and 7). Like the patient groups, both follow accounts within their own audiences at a higher rate than average. They both also follow their respective patient groups at higher than average rates. However, many pain specialists also follow members of the nonpharmacologic pain treatment audience, whereas cancer specialists do not often follow any kind of analogous group focused on nonpharmacologic cancer treatments.

While the SNAs have slightly different audience labels, both the Health Industry audience in the Pain SNA and the Oncology SNA’s Life Sciences audience include the pharmaceutical industry. Please note, however, that as the name reflects, the Life Sciences audience in the Oncology SNA includes a large group of researchers.

#### Comparative follower patterns—Comparative HCP specialists

The nonspecialist HCPs’ (Figs 6 and 7) relationships to the specialist follower patterns are more remarkable than the difference between those that appear on the Pain or Oncology SNAs. The nonspecialists within the Pain SNA appear to be less attentive to both patients and nonpharmacologic providers than the pain specialists. They also appear to be more likely to follow the Health Industry and Policy–News than the specialists. The nonspecialists in the Oncology SNA appear to be only slightly more likely to follow patients. However, the general HCPs appear to be more likely to follow oncology specialists than the general HCPs in the Pain SNA were to follow pain specialists (Figs 6 and 7). Members of the general HCPs audience within the Pain SNA appear to follow chronic pain patients somewhat less than general HCPs within the Oncology SNA follow cancer patients.

Unlike other audiences, the nonpharmacologic pain providers appear to be somewhat insular, in that they appear to be more likely to follow members within the same audience than all other audiences in the SNA (Fig 6). This pattern is a sign that this audience may be isolated from the rest of the SNA audiences—interestingly, this may be more of a self-reflection within this group rather than how others view them because other groups do follow them at substantial rates.

### 2. Identify what the similarities and differences in engagement among patients and HCPs within the Pain and Oncology SNAs tell us

As we examined the chronic pain SNA, we found that chronic pain patients follow each other at higher-than-average rates compared with a random mixing baseline, which suggests that they often get information from like-minded accounts; they also are more likely than oncology patients to cite consumer-focused information sources. They also appear to be engaged on general topics, such as pop culture, and topics related to nonpharmacologic pain treatment sources (Fig 6). In comparison, though cancer patients are equally likely to follow each other, they appear to be more likely to follow specialists than their chronic pain counterparts. They also cite health-focused sources such as jamanetwork.com and cancer.gov at higher rates than chronic pain patients.

### 3. Compare word use and citations among pain patients and HCPs

We then analyzed language use within the Pain SNA patient and provider audiences to gain further insights into the similarities and potential gaps between these audiences. In order to confirm that the initial analysis captured the pain conversation and to summarize the thousands of tweets that took place in the Pain SNA, the top 12,000 pairs of words were generated and ranked within each audience. Common word pairs that had no significance (i.e., pairs such as “good luck” and “happy birthday”) were removed, as were proper nouns.

While many words such as “health care” were used across the audiences, chronic pain patients were both focused on chronic pain and illness and most likely to use terms tied to feelings (i.e., pairs such as “feels like,” “looks like,” and “sounds like”). Specialist HCPs (both those involved in Pain Medicine and Nonpharmacologic Pain Treatment) had different lexicons in talking about pain. Table 1, the top word pairs used by the chronic pain patients, demonstrates that they were primarily discussing pain during the evaluated time period.

#### Comparative sources (other social media sites not included)

The information and media sources cited in audience members’ tweets indicate which of these sources have the most influence over each audience. Understanding the URL citations by each cluster cites, compared to the URL citations of other clusters, provides insight into the type of content shared on Twitter by pain patients and providers.

As not every tweet contains a URL citation, we expanded the collection period to nine months, which enabled the collection of 363,733 citations of 23,753 sources across all audiences in the Pain SNA, and then ranked which sources were used by each audience.

While both patients and HCPs often post stories from large, traditional media sources (for example, both most frequently cite nytimes.com), only HCPs cite sources related to health care, including sources such as statnews.com and khn.org (Kaiser Health News). Pain patients do not cite medical or health resources. The pain patients’ behavior of citing consumer sites, in combination with their focus on pain-related words within discussions, indicates that their online behavior appears to focus on pain, but they may not have adequate medically focused resources to cite. In Table 2, citations refer to the number of times the members of each audience mentioned the various Web domains on Twitter between July 2017 and March 2018.

### 4. Outline the challenges and limitations in addressing any differences between the audiences evaluated

The Twitter platform’s Streaming API precise sampling method is not disclosed. Therefore, while we were able to get full data on the following relationships’ specific tweets containing the language used, sources cited, and accounts mentioned analyses were only done based on that sampling. This may have impacted the citation ranking and our understanding of the language used.

Inclusion of users into audiences was based on similarity of sources they cite, and then the audience name was identified, which creates a limitation that some users within each audience might behave like that audience but not be a member of it. For example, a journalist who is focused on covering a specific audience might appear as a member of that audience.

As the focus of our research was to look at how the typical member of an audience interacted with other audiences, we normalized the data to account for audience size. Therefore, our findings are limited to how members of specific audiences act and not on how influential or active a specific audience is.

## Discussion

In this paper, we sought to identify the online relationships between pain patients and providers to understand what insights those relationships might provide. We found that on Twitter, pain patients and providers appear to interact less than oncology patients and providers. Pain patients do not appear to follow medical professionals or share medical or health-related information on Twitter to the same extent as oncology patients. In addition, we found that pain patients do not communicate on Twitter in the same language as HCPs. This is of interest because it reinforces a larger disconnect between pain patients and providers that has been well-documented in the literature [28]. It shows that challenges in communication are not just occurring in face-to-face interactions, but also in their digital social network (Twitter) interactions, serving as an additional roadblock to what can be shared decision-making opportunities around pain management.

Social media platforms are important tools for patient engagement and research has found that participating in online communities may improve health outcomes for certain conditions [29, 30]. Likewise, studies have examined how communication and team-based provider care can be essential for a person’s pain management plan [31].

Research has found that clear communication in a clinical setting is important for improved health outcomes in both pain patients [32] and oncology patients [33]. In fact, an article published by Thorne and Stajduhar identifies specific challenges that oncologists in Canada face when it comes to communicating with patients as they transition from primary cancer treatment to survivorship. The authors write:

“Although extensive empirical work informs communication in certain highly sensitive contexts such as ‘bad news’ delivery and the transition to palliative care, less is understood about communications in the course of routine care [34]”.

While our research did not focus on the clinical relationship, how patients and providers from these respective medical communities follow one another and communicate about pain differs significantly.

We provide some reasons why pain patient interactions on Twitter differ from those of pain providers: pain can be a part of many different conditions and, as a result, pain patients may not be organized online in the same way as other patients are, such as our oncology comparison; pain patients are also using a different vocabulary in their tweets than pain providers, which suggests that they may be viewing different sources of information than pain providers; in addition, pain patients might not have a central hub of evidence-based information to cite, such as a leading scientific and patient-friendly resource like cancer.gov. Research has also found that while the internet can be a place where pain information is found in abundance, at present, it is not well-used as a space for patient‒provider interactions [35]. Our results, in addition to these potential explanations, point toward a need for an online, evidence-based resource hub that could benefit the pain patient community in the same way that cancer.gov serves as a resource of aggregated materials, information to bring to HCPs, studies, and content from many cross-sections of oncology.

More research is needed to explore how an online analysis can translate to face-to-face physician/patient interactions and if the tools and outreach materials developed to close the engagement and language gap are improving health outcomes in people with pain. A comparative analysis using the same methodology could be conducted after additional resources are developed and deployed to see if online behavior is impacted. At the same time, traditional survey research could be used to determine whether similar patterns of behavior exist for patients that are not engaged with social media.

## Supporting information

S1 File Figure data & notes. (DOCX)

S2 File NCCIH pain campaign literature review. (DOCX)

S3 File NCCIH pain campaign media audit. (DOCX)

S4 File Glossary. (DOCX)

S5 File Pain and oncology SNA Twitter users. (XLSX)
