# Supplementary material for: What Twitter teaches us about patient-provider communication on pain
Source: PLoS One. 2019 Dec 26;14(12):e0226321. doi: 10.1371/journal.pone.0226321 (PMC6932781; doi:10.1371/journal.pone.0226321)
Supplement: S1 File — (DOCX) [file pone.0226321.s001.docx]

**S1 File**

**Figure Data & Notes**

**S1 Table 1**

**Fig 1 Details: Groups and Clusters**

| **Cluster Name** | **Label** | **Group Name** |
| --- | --- | --- |
| Arthritis | clu_27 | Chronic Pain Patients |
| Chronic Conditions | clu_4 | Chronic Pain Patients |
| Engaged Patients | clu_5 | Chronic Pain Patients |
| Migraines | clu_42 | Chronic Pain Patients |
| Neurological Patients | clu_26 | Chronic Pain Patients |
| Pain Patient Advocates | clu_3 | Chronic Pain Patients |
| Pain Support - Women Centric | clu_14 | Chronic Pain Patients |
| Patient Advocacy | clu_36 | Chronic Pain Patients |
| RSD-CRPS | clu_45 | Chronic Pain Patients |
| Spoonies | clu_17 | Chronic Pain Patients |
| Australian Pain | clu_29 | Culture/Other |
| Health Celebrities | clu_1 | Culture/Other |
| Unclustered | clu_0 | Culture/Other |
| Addiction Recovery | clu_25 | HCP's |
| Anesthesiology | clu_7 | HCP's |
| Digital Health Innovators | clu_28 | HCP's |
| Family Physicians | clu_12 | HCP's |
| Health Providers | clu_22 | HCP's |
| Hospitalists | clu_37 | HCP's |
| Hospitals | clu_11 | HCP's |
| Hospitals HealthSystems | clu_19 | HCP's |
| Mental Health | clu_41 | HCP's |
| Neurology | clu_13 | HCP's |
| Nursing | clu_2 | HCP's |
| Oncology | clu_23 | HCP's |
| Palliative Care | clu_43 | HCP's |
| Physicians | clu_18 | HCP's |
| Senior Care | clu_32 | HCP's |
| Surgery | clu_21 | HCP's |
| BioPharma | clu_40 | Health Industry |
| Health IT | clu_16 | Health Industry |
| Integrative Aproaches | clu_9 | Nonpharmacologic Pain Treatment |
| Physiotherapy | clu_39 | Nonpharmacologic Pain Treatment |
| Physiotherapy - Spain | clu_15 | Nonpharmacologic Pain Treatment |
| PT & Spine | clu_33 | Nonpharmacologic Pain Treatment |
| PT & Sports | clu_44 | Nonpharmacologic Pain Treatment |
| Pain Management | clu_8 | Pain Medicine & Research |
| Pediatric Pain | clu_46 | Pain Medicine & Research |
| UK Pain Medicine | clu_10 | Pain Medicine & Research |
| Health Journalists | clu_34 | Policy - News |
| Health Policy | clu_35 | Policy - News |
| Health Policy Focus | clu_20 | Policy - News |
| Health Policy News | clu_38 | Policy - News |
| Healthcare News | clu_30 | Policy - News |
| Public & Global Health | clu_24 | Public Health |
| Public Health | clu_6 | Public Health |
| Public Health - Gov Focused | clu_31 | Public Health |

**S1 Table 2**

**Fig 4 Details: Groups & Clusters**

| **Cluster Name** | **Label** | **Group Name** |
| --- | --- | --- |
| Patient Advocacy | clu_23 | Cancer Patient Advocacy |
| Inherited / Breast Cancer Focused | clu_2 | Cancer Patient Advocacy |
| Pediatric Cancer | clu_1 | Cancer Patient Advocacy |
| Melanoma Advocacy | clu_35 | Cancer Patient Advocacy |
| Cancer Support | clu_6 | Cancer Patient Advocacy |
| Lung Cancer | clu_4 | Cancer Patient Advocacy |
| Canadian | clu_42 | Cancer Patient Advocacy |
| Cancer Advocacy | clu_7 | Cancer Patient Advocacy |
| Gynecologic Cancers | clu_3 | Cancer Patient Advocacy |
| Health Inspiration | clu_22 | Digital / Social Media |
| Health Social Media | clu_9 | Digital / Social Media |
| Health IT | clu_19 | Digital / Social Media |
| Health Disruption / Digital | clu_15 | Digital / Social Media |
| Health Social Marketing | clu_12 | Digital / Social Media |
| General Health | clu_0 | Health News / Policy |
| Celebrities - Pop Culture | clu_13 | Health News / Policy |
| News / Policy | clu_11 | Health News / Policy |
| General Health | clu_28 | Health News / Policy |
| Popular Health \| \|Nutrition | clu_29 | Health News / Policy |
| Health News / Journalism | clu_39 | Health News / Policy |
| US Politics | clu_30 | Health News / Policy |
| Genomics | clu_17 | Life Sciences |
| BioPharma Industry | clu_24 | Life Sciences |
| BioPharma / Health Tech Focus | clu_47 | Life Sciences |
| Science News | clu_16 | Life Sciences |
| BioPharma Research | clu_5 | Life Sciences |
| Science Writers / Journalists | clu_45 | Life Sciences |
| BioPharma News | clu_20 | Life Sciences |
| Science / Genomic Focused | clu_32 | Life Sciences |
| Rare / Genetic Diseases | clu_26 | Life Sciences |
| Radiology | clu_44 | Non-Oncology HCP / Hospitals |
| Hospital Focused | clu_27 | Non-Oncology HCP / Hospitals |
| Palliative / Hospice | clu_8 | Non-Oncology HCP / Hospitals |
| Cardiology | clu_40 | Non-Oncology HCP / Hospitals |
| Hospitals & Health Systems | clu_36 | Non-Oncology HCP / Hospitals |
| Internal Medicine | clu_18 | Non-Oncology HCP / Hospitals |
| Surgery | clu_41 | Non-Oncology HCP / Hospitals |
| Nursing | clu_21 | Non-Oncology HCP / Hospitals |
| Australian | clu_31 | Oncology HCP |
| Cancer Treatment | clu_34 | Oncology HCP |
| Hematology / Oncology | clu_10 | Oncology HCP |
| Medical Oncology | clu_14 | Oncology HCP |
| Pathology | clu_33 | Oncology HCP |
| British | clu_37 | Oncology HCP |
| Urology / Oncology | clu_43 | Oncology HCP |
| Global Health | clu_46 | Public Health |
| Public Health - US Gov Focused | clu_38 | Public Health |
| Public Health | clu_25 | Public Health |

**S1 Table 3**

**Fig 6 Data: Who do pain audiences follow?**

**Chronic Pain Patients**

| Audience | Total Follows by Chronic Pain Audience | Normalization: Follow Density in Relation to Audience Size | Follow Percentage after Normalization |
| --- | --- | --- | --- |
| Chronic Pain Patients  (Total members: 2141) | 266,961 | 0.058 | 36% |
| Pain Medicine & Research  (Total members: 787) | 25,056 | 0.015 | 9% |
| HCP's  (Total members: 3655) | 78,974 | 0.010 | 6% |
| Nonpharmacologic Pain Treatment  (Total members: 1177) | 27,819 | 0.011 | 7% |
| Culture/Other  (Total members: 597) | 2,9832 | 0.023 | 14% |
| Health Industry  (Total members: 846) | 12,355 | 0.007 | 4% |
| Policy – News  (Total members: 1119) | 17,508 | 0.007 | 4% |
| Public Health  (Total members: 1764) | 120,556 | 0.032 | 20% |

**Pain Medicine & Research**

| Audience | Total Follows by Pain Medicine & Research Audience | Normalization: Follow Density in Relation to Audience Size | Follow Percentage after Normalization |
| --- | --- | --- | --- |
| Chronic Pain Patients  (Total members: 2,141) | 31,518 | 0.019 | 16% |
| Pain Medicine & Research  (Total members: 787) | 22,300 | 0.0369 | 31% |
| HCP's  (Total members: 3,655) | 27,349 | 0.010 | 8% |
| Nonpharmacologic Pain Treatment  (Total members: 1,177) | 16,335 | 0.018 | 15% |
| Culture/Other  (Total members: 597) | 5,359 | 0.011 | 10% |
| Health Industry  (Total members: 846) | 3,157 | 0.005 | 4% |
| Policy – News  (Total members: 1,119) | 4,143 | 0.005 | 4% |
| Public Health  (Total members: 1,764) | 19,099 | 0.014 | 12% |

**HCPs**

| Audience | Total Follows by General HCPs - Pain Audience | Normalization: Follow Density in Relation to Audience Size | Follow Percentage after Normalization |
| --- | --- | --- | --- |
| Chronic Pain Patients  (Total members: 2,141) | 59,865 | 0.008 | 4% |
| Pain Medicine & Research  (Total members: 787) | 20,409 | 0.007 | 4% |
| HCP's  (Total members: 3,655) | 593,299 | 0.044 | 24% |
| Nonpharmacologic Pain Treatment  (Total members: 1,177) | 21,820 | 0.005 | 3% |
| Culture/Other  (Total members: 597) | 33,036 | 0.015 | 8% |
| Health Industry  (Total members: 846) | 120,777 | 0.039 | 21% |
| Policy – News  (Total members: 1,119) | 126,493 | 0.031 | 16% |
| Public Health  (Total members: 1,764) | 246,480 | 0.038 | 20% |

**Nonpharmacologic Pain Treatment**

| Audience | Total Follows by Nonpharmacologic Pain Treatment Audience | Normalization: Follow Density in Relation to Audience Size | Follow Percentage after Normalization |
| --- | --- | --- | --- |
| Chronic Pain Patients  (Total members: 2,141) | 15,066 | 0.006 | 7% |
| Pain Medicine & Research  (Total members: 787) | 8,888 | 0.010 | 11% |
| HCP's  (Total members: 3,655) | 24,695 | 0.006 | 6% |
| Nonpharmacologic Pain Treatment  (Total members: 1,177) | 65,366 | 0.047 | 52% |
| Culture/Other  (Total members: 597) | 3,842 | 0.005 | 6% |
| Health Industry  (Total members: 846) | 3,595 | 0.004 | 4% |
| Policy – News  (Total members: 1,119) | 4,224 | 0.003 | 4% |
| Public Health  (Total members: 1,764) | 18,917 | 0.009 | 10% |

**S1 Table 4**

**Fig 7 Data: Who do cancer audiences follow?**

**Cancer Patient Advocacy**

| Audience | Total Follows by Cancer Patient Advocacy Audience | Normalization: Follow Density in Relation to Audience Size | Follow Percentage after Normalization |
| --- | --- | --- | --- |
| Cancer Patient Advocacy  (Total members: 1,704) | 415,618 | 0.143 | 42% |
| Oncology HCP  (Total members: 1,489) | 147,574 | 0.058 | 17% |
| Non-Oncology HCP/Hospitals  (Total members: 1,590) | 57,557 | 0.021 | 6% |
| Digital/Social Media  (Total members: 1,984) | 129,348 | 0.038 | 11% |
| Health News / Policy  (Total members: 2,277) | 143,839 | 0.037 | 11% |
| Life Sciences  (Total members: 2,377) | 72,489 | 0.018 | 5% |
| Public Health  (Total members: 1,196) | 52,671 | 0.026 | 8% |

**Oncology HCPs**

| Audience | Total Follows by Oncology HCPs Audience | Normalization: Follow Density in Relation to Audience Size | Follow Percentage after Normalization |
| --- | --- | --- | --- |
| Cancer Patient Advocacy  (Total members: 1,704) | 146,352 | 0.058 | 18% |
| Oncology HCP  (Total members: 1,489) | 227,821 | 0.103 | 32% |
| Non-Oncology HCP/Hospitals  (Total members: 1,590) | 73,379 | 0.031 | 10% |
| Digital/Social Media  (Total members: 1,984) | 83,180 | 0.028 | 9% |
| Health News / Policy  (Total members: 2,277) | 115,259 | 0.034 | 11% |
| Life Sciences  (Total members: 2,377) | 111,904 | 0.032 | 10% |
| Public Health  (Total members: 1,196) | 59,812 | 0.034 | 11% |

**Non-Oncology HCPs/Hospitals**

| Audience | Total Follows by Nononcology HCPs/Hospitals Audience | Normalization: Follow Density in Relation to Audience Size | Follow Percentage after Normalization |
| --- | --- | --- | --- |
| Cancer Patient Advocacy  (Total members: 1,704) | 57,443 | 0.021 | 6% |
| Oncology HCP  (Total members: 1,489) | 75,621 | 0.032 | 9% |
| Non-Oncology HCP/Hospitals  (Total members: 1,590) | 293,482 | 0.116 | 31% |
| Digital/Social Media  (Total members: 1,984) | 191,457 | 0.061 | 16% |
| Health News / Policy  (Total members: 2,277) | 173,104 | 0.048 | 13% |
| Life Sciences  (Total members: 2,377) | 78,605 | 0.021 | 6% |
| Public Health  (Total members: 1,196) | 130,732 | 0.069 | 19% |
