# Supplementary material for: What Twitter teaches us about patient-provider communication on pain
Source: PLoS One. 2019 Dec 26;14(12):e0226321. doi: 10.1371/journal.pone.0226321 (PMC6932781; doi:10.1371/journal.pone.0226321)
Supplement: S2 File — (DOCX) [file pone.0226321.s002.docx]

**S2 File**

Date: September 9, 2016

To: National Center for Complementary and Integrative Health (NCCIH) OCPL

From: JPA Health Communications

Re: NCCIH Pain Campaign Literature Review

Overview

As part of formative research on a pain-focused campaign for the National Center for Complementary and Integrative Health (NCCIH), JPA conducted a literature review to form an understanding of the needs of patients and providers in addressing chronic pain with informed, evidence-based decision-making.

This review evaluated the following:

- Existing assets and research, as well as gaps, in the current evidence base for communication around complementary approaches for pain management
- Key themes within existing research
- Implications and opportunities for an NCCIH pain communication campaign

*A note on scope: The previous NCCIH public campaign, “Time to Talk,” focused on the importance of initiating patient-provider conversations about complementary approaches. We anticipate the next NCCIH campaign will broaden in scope to support not just patient-provider conversations, but also consumers using complementary approaches for self-management of pain. However, given physicians’ reliance on academically robust research and the urgent need to equip primary care providers and their chronic pain patients with options in addition to opioids or other pharmacologic treatments, patient-provider interactions are a key focus of this literature review. Other formative research assets will be more focused on patients themselves and their attitudes.*

*A note on definitions: “Chronic pain” is not broken down in most of the literature reviewed here in terms of associated health conditions. As a public health phenomenon, it tends to be discussed with reference to impact on function and not to underlying cause. Exceptions: one paper reviewed mentioned chronic cancer pain and another mentioned osteoarthritis. A separate Media Audit and Audience Tracker analysis will help to further break down conditions associated with chronic pain. “Provider” typically refers here to primary care providers.*

Key Findings

In our assessment, the literature suggests a need for more effective communication about pain between chronic pain patients and their providers. Current research on pain communication shows that there are a number of unmet needs for patients and providers discussing pain and treatment options; each group faces challenges that limit communication. Some of these are mutual challenges, such as a lack of shared vocabulary. However, others are specific to each group, such as patients’ need for providers to show more acceptance toward narrative descriptions of the pain experience and its impact. Support for development and sharing of such narratives would improve patients’ confidence in learning about, performing and tracking self-management of pain outside of clinical settings; it also would help communication efforts with other patients and integrate discussion of self-management into provider communication. Finding space for patient pain narratives in treatment settings, though challenging due to lack of time, would also improve communication between patients and providers at all stages of the pain management process.

There are only limited resources available from authoritative sources, such as guidelines from federal health agencies, regarding the safety and efficacy of complementary approaches and effective integration of complementary approaches into pain management via patient-provider conversations. Current American Medical Association (AMA)-validated CME courses on nonpharmacologic options for chronic pain do not go into great depth about evidence-based options and do not focus on patient-provider interaction around complementary health approaches for pain.

There is little guidance in existing research about using complementary and integrative approaches specifically for pain management. Complementary modalities are mentioned as plausible options combined with or as alternatives to pharmacologic approaches, but information on their efficacy for painful conditions and guidance on recommending them to patients for treatment or self-care was rarely included in guidelines or other communications from medical organizations and health agencies to providers about patient interaction.

A few key themes emerged during this review of relevant articles:

- In the literature, self-management techniques emerge as one of the more effective approaches to help patients handle pain.
- Despite this, programs promoting self-management techniques within patient populations lag in implementation, according to the [National Pain Strategy](https://iprcc.nih.gov/National_Pain_Strategy/NPS_Main.htm) released by the HHS Interagency Pain Research Coordinating Committee (IPRCC) earlier this year.
- Studies also touched on the importance of patient-provider communication and the role of emotion, feelings and perception in the way pain management information is interpreted and used for decision-making.

These themes are often intertwined in the literature, and in developing the campaign we will continue to deepen our understanding of their impact on effective use of evidence-based complementary approaches in pain management.

Methodology

To conduct this review, JPA retrieved 40 articles related to communication by and with patients about pain management, their priorities for pain treatment and the information they can access about it, using search terms including “pain information communication,” “pain management communication,” “pain patient communication,” “pain management information” and “pain patient priorities.” The team examined articles from various fields of study including health services research, health communications, medical practice research, behavioral and cognitive science, complementary and integrative health research and federal documents. The following filters were applied to the initial longlist:

- Relevant to patient populations in the United States (primarily), Canada, the United Kingdom or Australia (secondarily)
- Published between 2013 and 2016 (for non-federal documents)
- Peer-reviewed or otherwise of outstanding credibility, for example authorship by a federal health agency or validation by a respected medical professional organization, such as the AMA
- High quality study design per best practice standards for respective disciplines
- Potential to illuminate alignment or difference within the pain management community about communication of information

Each of the 14 articles in the resulting shortlist for inclusion was considered within the context of key questions documented in the formative research approach proposal (separate document) and reviewed for insight that could help shape a public health communication campaign around complementary and integrative interventions for pain.

Review of Existing Research – What We Found

Tailoring pain treatment plans for patients poses a difficult clinical challenge. Providers have a duty to help their patients manage pain and not to be defined by it (Chang, 2016). Though providers have some information resources for navigating pain management decisions, they face difficulties in applying them consistently, because individual patients’ pain experiences vary greatly. The research provided a view of the current clinical context for – and challenges to providers in creating – efficient, effective pain treatment plans:

- The levels of pain experienced by patients do not always correlate with physical pathology for chronic conditions (Franklin, 2016). This can prove difficult for providers, who may see patients with the same condition experience vastly different pain severities.
- Clinicians use many mechanisms to measure the individual pain experience beyond simply looking at its intensity. Pain can be analyzed by considering the extent to which it disrupts daily living, work and function (IOM, 2011).
- Clinicians typically follow guidelines for pain treatment, but they do not always consider their patients’ preferred pain management strategies (Franklin, 2016).
- Patients, who physically endure pain and risk feeling helpless in their search for relief, are often not told or do not understand that the road to finding the right combination of approaches may be long (IOM, 2011).
- Patients have unique perceptions of their own bodies, minds, and wellness that come into play at all stages of the pain management process, from consultation to implementation of a treatment regimen (Neeley, 2016).

Individual patient experiences vary greatly, making it difficult to both conduct research on pain experience and treat it. When navigating effective treatment approaches for pain, there are a number of social, biological, and psychological factors at play (Driscoll, 2016). Some studies in this review showed the power of emotions and expectations in treatment settings.

Research suggests consistencies in the way emotion affects decision making. This clarifies the importance of understanding how a person’s feelings may affect how he interprets pain management information and uses that information to make decisions. Integral emotions related to a situation at hand tend to beneficially guide decisions and limit risk-taking, while other emotions carried into a situation from unrelated areas of life also have the potential to affect decisions (Lerner, Li, Valdesolo, & Kassam, 2015). We can infer that it is helpful for patients to engage in narrative-building around their pain experience, to help both themselves and their providers identify factors that may be at play in their pain experience. Storytelling can be a useful tool to create meaning out of events (Neeley, 2016).

Expectations and how they are conveyed also play key roles in the individual patient experience of complementary and other therapies for pain. A study that looked at the expression of expectations within treatment found that clinicians who communicate more optimism for success may enhance the patient experience during a period of five to six weeks of treatment – and with regard to a specific complementary modality, clinicians who communicated higher, not neutral, expectations saw patients with osteoarthritis pain were more satisfied with acupuncture four weeks into treatment (Street, 2013). Though more research is necessary to understand the role expression and shared language play in patient‒provider communication and potential placebo effects, the literature suggests there is a link between language and sentiment in these interactions and pain management outcomes.

Though there are resources available to providers on effective complementary and integrative approaches for pain, these approaches are still not perceived as “go-to” treatments. However, credible clinical resources emphasize self-management programs, which can include complementary approaches.

The discussion of complementary and integrative approaches for chronic pain in this research helped create a sense of how they are perceived by healthcare professionals working in pain management. Some guidelines provide a look at how complementary approaches are discussed in clinical practice. Other research sheds light on effective channels for delivering pain management information to patients.

Based on a review of the AMA-approved Continuing Medical Education (CME) resource on complementary therapies in chronic pain treatment, current clinical practice is to standardize care from within a medical home, then move forward to individualize care and consider complementary approaches (Saenger & Drexler, 2013). The CME held additional insights relative to complementary approaches and how they are perceived by clinicians:

- Perhaps unhelpfully, the term “CAM” is still used and meant to be all-encompassing of “all things ‘outside the box’ of Bio-Medicine.”
- These specific guidelines note the potential for acupuncture, massage, spinal manipulation, progressive relaxation and yoga to help people with chronic pain, whereas prolotherapy and herbal remedies were deemed unlikely to help.
- Providers are encouraged to ask patients which approaches they have tried or would like to try, keeping in mind the limitations and benefits of all therapies and the potential for a package of step-by-step options.

Additional key findings on pain management approaches in practice include:

- Self-management programs can improve quality of life and are an important component of acute and chronic pain prevention and management (National Pain Strategy, 2016).
  - Research also suggests these programs can assist the management of musculoskeletal pain and depression – and in the process may help providers communicate more efficiently with patients about pain (Damush, 2016).
- A shared approach to medical decision making, potentially using the SMART framework, was considered helpful for patients in establishing pain management goals. A stepped care model for pain, which first delivers the most effective yet least resource-intensive treatment, is also considered beneficial (Driscoll, 2016).
- Technological advances and eHealth can play key roles in comprehensive pain management plans.
  - Collaborative management interventions such as “telecare” or automated symptom monitoring have been shown to help improve patients’ pain and lower the likelihood of an increase in opioid dosage (Kroenke, Krebs, Wu, Chumbler, & Bair, 2014).

For any management plan, guidelines call upon providers to consider each intervention’s safety, clinically important differences in effect, and impact on resource stewardship (Saenger & Drexler, 2013).

The evidence base for opioid use in chronic pain is not much more robust than that for complementary approaches.

The risk of using pharmacologic approaches such as opioids for long-term pain treatment was frequently cited in this research, though many chronic pain patients rely on them for long-term pain amelioration (a topic that will be covered in a separate Media Audit). Current guidelines implore patients and providers to discuss the risks associated with long-term opioid therapy such as opioid use disorder, overdose and death (National Pain Strategy, 2016). The evidence base for opioid use in chronic pain is weak and not much more robust than that for complementary approaches, though this is often unclear or even unknown to patients seeking relief (Saenger & Drexler, 2013).

Guidelines also recommend consideration of a combination of factors when assessing the clinical effectiveness of any therapy, whether conventional, complementary or usual care:

- These include outcomes such as the effect of the intervention on pain, function and side effects along with associated direct and indirect costs (Saenger & Drexler, 2013).
- It is recommended that physicians integrate elements of a cognitive behavioral approach into practice, giving patients an active role in the treatment plan and supporting them in taking on beneficial but potentially anxiety-provoking approaches, such as exercise (Saenger & Drexler, 2013).

Sensitivity to patients’ individual experiences of pain is as important as it is difficult to practice.

Providers must be vigilant in their treatment plans to recognize aberrant use of opioids but also remain sensitive to patient reports of pain, keeping the element of individual experience and the invisibility of pain severity in mind (Chang, 2016). Unlike some more “cut and dried” conditions that improve with specific treatment, it’s often impossible for providers to predict which treatment or combination of treatments will work best to help manage an individual’s pain (IOM, 2011).

This reality is complicated by a treatment process that is often more prescriptive than consultative, as many physicians face packed schedules and other factors that severely limit time for connecting with patients to understand their pain and tailor a treatment plan (Franklin, 2016). Although not stated specifically, these documented challenges may show there are too few opportunities to engage in sensitive, narrative-driven, patient-led interactions about pain.

Public understanding of scientific information is limited, which affects patient-provider conversations and, as a result, decisions about pain treatment.

There is still a need for more studies on effective patient-provider communication and decision making. It is important to keep in mind that individuals must often grapple with imperfect or limited evidence when making decisions about their care and may simultaneously struggle with differentiating between high and low quality evidence (Drummond, 2016). Limited understanding of how to decipher scientific information can affect patients’ decisions about their pain treatment – a hurdle for providers in using current guidelines, resources and research within a collaborative management process.

A study examining uncertainty about pain management within analyses of patient and Veteran’s Affairs primary care provider interaction around opioids for chronic pain identified three patterns of response:

1. Reassurance: When the provider educates or expresses concern about the patient’s opioid use, the patient’s typical response is that he is not “abusing” the medication.
2. Avoidance: Frequently the provider and patient discuss avoiding opioid use. One patient was quoted saying “I’m trying to stay off narcotics. I don’t want to get addicted.’’ Providers tend to express concerns about long-term opioid use.
3. Gathering additional information: When the patient had a history of substance abuse, visits often include candid discussions about opioids and even urine drug screens to test for recent drug use (i.e., cocaine).

Though the study showed the importance of candid conversations between patients and providers, it also concluded that more exploration is needed to support design of methods to improve patient-provider communication about opioid treatment (Matthias, 2013).

There are resources available to help providers communicate the risks and benefits of pain management options to patients. They range from key practice recommendations in publications such as the American Academy of Family Physician’s *FP Essentials* to a standardized definition of interdisciplinary pain care such as that from the International Association for the Study of Pain. A webinar describing methods used to develop American Pain Society and American Academy of Pain Medicine guidelines for using chronic opioid therapy in chronic noncancer pain shares recommendations for that area of pharmacologic treatment and current research that may affect prescribing (Clinical Guidelines for Opioid Use in Chronic Noncancer Pain Archive Webinar & Post-test, 2015). What is unclear, following this research, is which resources currently serve as favored or “go to” sources for information on pain treatment for patients and providers.

This body of research helps us identify where NCCIH can fill the gaps in patient and provider understanding of complementary and integrative approaches for chronic pain conditions and equip both parties with the information they need to facilitate productive conversations relevant to an integrated management plan.

Implications and Opportunities for an NCCIH Campaign

It is clear that NCCIH information can help play a key role in helping patients and health care professionals more effectively leverage self-management techniques for pain. This review sheds light on the difficulties faced by both sides:

- Patients experiencing the physical and emotional burden of pain, the frustration of searching for the right combination of approaches for treatment, struggling to articulate pain experiences in ways that make them feel heard by their providers.
- Patients working with limited knowledge of science, providers limited by outdated or mistaken perceptions of the evidence base in pain management and both groups limited by the relatively small evidence base for complementary approaches in chronic pain management – as well as a lack of resources to facilitate patient-provider communication about these issues.
- Providers working within various guidelines, running the risk of burnout and lacking adequate time for effective one-on-one patient communication.

The importance of better facilitating discussion between providers and patients on complementary and integrative interventions is evident. Though clear guidelines and resources aid the development of treatment plans, more shared language around pain experiences and support for narrative-driven patient accounts of pain would also be helpful. When stories of pain are taken seriously, all involved parties are able to bear witness to the toll of pain on human lives, work to understand its root causes and work toward its alleviation (Neeley, 2016).

A campaign cannot solve the opioid addiction epidemic and complementary approaches are not going to replace opioids. It is not our role to divert patients from primary care providers toward completely independent self-care; rather, we will support clinical practice AND self-care by positioning complementary approaches as accessible first-line tools within a patient-centered approach to pain treatment.

NCCIH research and resources can inform the development of helpful tools for beneficial conversations between patients and physicians, remaining empathetic to both sides of the discussion and supporting the development of shared language about pain experiences. They can also introduce or increase the understanding of complementary approaches among patients and providers and add context to recommendations regarding nonpharmacologic approaches to pain management. NCCIH may also explore how a campaign could help providers shift their pain treatment process from prescriptive to consultative when faced with time restrictions.

Audience:

- Chronic pain patients with noncancer-related pain; mainly middle-aged and older adults
- Providers, with a focus on primary care

Problem: It is challenging for patients and providers to engage in consultative discussions around pain management. A more consultative approach, bolstered by evidence-based resources, will allow for more personalized care that integrates complementary and integrative approaches with pharmacologic approaches.

Gaps:

- There is a lack of shared language in the clinical setting, which can lead to miscommunication, frustration on both sides and patient hesitance to seek alternative treatment
- Physicians lack adequate time for consultation with patients
- The evidence base for complementary and integrative approaches is still growing, and these approaches are not perceived as “go-to” treatments for use alongside traditional care
- There are limited resources available from authoritative sources regarding the safety and efficacy of complementary approaches and ways to effectively integrate them into pain management.

Next Steps

This literature review is the first step in the formative research process for the NCCIH campaign. It has helped answer a number of key questions necessary to craft a thoughtful campaign around treatment and communication regarding pain.

A few key questions the literature review might have addressed were not covered by the identified articles. The following will be investigated in other pieces of formative research:

- What delivery channels might be most effective in providing pain management to consumers?
- What resources do consumers use for information on complementary health approaches?
- What resources do providers use for information on complementary health approaches? (answered in part but will also be addressed by other formative research)

Additionally, to reiterate, the voice of the chronic pain patient is critical to any future campaign and will be addressed in other elements of formative research – but was not a strong element of the literature review.

JPA is also continuing work on the media audit, which will identify relevant themes in top-tier consumer media outlets, patient and provider blogs, federal health agency blogs, professional society newsletters and other relevant sources to address the media-related questions outlined in the formative research plan. The team has also created “issue bundles” of terms relevant to pain management, chronic pain, nonpharmacologic approaches to pain and complementary and integrative pain interventions to produce a campaign-focused Audience Tracker report that will help identify themes and stakeholders.

Bibliography

Chang, K. F. (2016). Chronic pain management: Nonpharmacological therapies for chronic pain. FP Essent. 2015 May;432:21-6.

Chou R, Fanciullo GJ, Fine PG, et al. Clinical Guidelines for the Use of Chronic Opioid Therapy in Chronic Noncancer Pain. The journal of pain : official journal of the American Pain Society. 2009;10(2):113-130. doi:10.1016/j.jpain.2008.10.008.

Damush, T. (2016). Pain self-management training increases self-efficacy, self-management behaviours and pain and depression outcomes. Eur J Pain. 2016 Aug;20(7):1070-8. doi: 10.1002/ejp.830. Epub 2016 Feb 5.

Driscoll, M. (2016). Integrated, Team-Based Chronic Pain Management: Bridges from Theory and Research to High Quality Patient Care. Adv Exp Med Biol. 2016;904:131-47. doi: 10.1007/978-94-017-7537-3_10.

Drummond, C. (2016). Scientific Reasoning Ability and its Implications for Science Communication. White paper prepared for University of Michigan conference on Health and Science Communication. Accessible from: https://www.isr.umich.edu/cps/events/Drummond_20160610.pdf.

Franklin, Z. C. (2016). A qualitative investigation of factors that matter to individuals in the pain management process. Disabil Rehabil. 2016 Sep;38(19):1934-42. doi: 10.3109/09638288.2015.1107782. Epub 2016 Jan 4.

IOM. (2011). Relieving Pain in America: A Blueprint for Transforming Prevention, Care, Education, and Research. Institute of Medicine (US) Committee on Advancing Pain Research, Care, and Education. Washington (DC): National Academies Press (US); 2011. The National Academies Collection: Reports funded by National Institutes of Health.

Kroenke, K., Krebs, E., Wu, J., Chumbler, N., & Bair, M. (2014). Telecare Collaborative Management of Chronic Pain in Primary Care: A Randomized Clinical Trial. JAMA. 2014 Jul 16;312(3):240-8. doi: 10.1001/jama.2014.7689.

Lerner, J. S., Li, Y., Valdesolo, P., & Kassam, K. (2015). Emotion and Decision Making. Annu. Rev. Psychol. 2015. 66:799–823 doi: 10.1146/annurev-psych-010213-115043.

Matthias, M. (2013). "I'm not Abusing or Anything": Patient-physician communication about opioid treatment in chronic pain. Patient Educ Couns. 2013 Nov;93(2):197-202. doi: 10.1016/j.pec.2013.06.021. Epub 2013 Aug 2.

National Pain Strategy. (2016). U.S. Department of Health and Human Services, Office of the Assistant Secretary for Health. Accessible from: https://iprcc.nih.gov/sites/default/files/HHSNational_Pain_Strategy_508C.pdf.

Neeley, L. (2016). The Value of Storytelling in Public Health and Medicine. White paper prepared for University of Michigan conference on Health and Science Communication. Accessible from: https://www.isr.umich.edu/cps/events/Neely_20160613.pdf.

Saenger, M., & Drexler, K. (2013, January 25). Doc, What Else Can I Do? Learning the Evidence Behind Complementary and Alternative Chronic Pain Management. APA PCSS-O. Accessible from: https://pdfs.semanticscholar.org/presentation/173e/8ee338abcc10982f671823c98766e2e6c4ca.pdf.

Street, R. L. (2013). How clinician–patient communication contributes to health improvement: Modeling pathways from talk to outcome. Patient Educ Couns. 2013 Sep;92(3):286-91. doi: 10.1016/j.pec.2013.05.004. Epub 2013 Jun 6.
