# Supplementary material for: What Twitter teaches us about patient-provider communication on pain
Source: PLoS One. 2019 Dec 26;14(12):e0226321. doi: 10.1371/journal.pone.0226321 (PMC6932781; doi:10.1371/journal.pone.0226321)
Supplement: S3 File — (DOCX) [file pone.0226321.s003.docx]

**S3 File**

Date: September 12, 2016

To: National Center for Complementary and Integrative Health (NCCIH) OCPL

From: JPA Health Communications

Re: NCCIH Pain Campaign Media Audit

Overview & Methodology

Media coverage can provide insight into the experience of living with or treating chronic pain. It provides a platform for patients to share their stories, a space for physician perspectives on current treatment practice and a sounding board for other stakeholder perspectives, guidelines and protocols.

This media audit is the second piece of formative research designed to help identify opportunities for a pain-focused campaign for the National Center for Complementary and Integrative Health. The first piece of research, a literature review, evaluated existing studies and assets in the current evidence base for communication related to pain management and complementary approaches. That process shed light on a number of questions that are key to crafting a thoughtful campaign, as well as implications and opportunities for an NCCIH campaign to fill gaps in patient‒provider communication regarding pain.

Through this media audit, JPA aimed to identify relevant themes and tap into current coverage of chronic pain treatment and related conversations. To conduct this review, the team identified 157 relevant articles from a variety of sources via specific search terms in Google News, routine article scanning and previous media monitoring reports. All articles were published between January 1 and August 31, 2016 and were reviewed to determine which key questions they could help answer. These analyses are available in the accompanying PDF of an Excel document.

*Please note that due to the time period, this review did not include coverage of NCCIH’s Mayo Clinic Proceedings paper,* [*Evidence-Based Evaluation of Complementary Health Approaches for Pain Management in the United States*](https://www.ncbi.nlm.nih.gov/pubmed/27594189) *(Mayo Clin Proc. 2016 Sep;91(9):1292-306. doi: 10.1016/j.mayocp.2016.06.007)*. However key insights from this paper were taken into consideration as the team reviewed coverage for this media audit.

*A note on definitions:* *The key questions guiding formative research for this campaign have been slightly revised to exclusively use “patients” rather than “consumers” and “providers” rather than “HCPs.” In contrast to the literature review, “providers” in this analysis refers to general practitioners / primary care providers AND to other providers, such as pain specialists. Most media coverage does not distinguish between provider categories.*

| **Search Terms** | |
| --- | --- |
| “pain community”  “pain” AND “primary care”  “pain” AND “complementary” OR “integrative”  “nonpharmacologic” AND “pain” OR “pain management”  “pain” AND “alternative” OR “alternative management”  “chronic pain”  “chronic pain” AND “opioids”  “veterans” AND “chronic pain” | “pain” AND “prescribing”  “pain” AND “challenges”  “pain” AND “self care” OR “self-care”  “pain” AND “talk” OR “conversation”  “National Pain Strategy”  “pain” AND “resilience”  “pain” AND “resources”  “pain” AND “provider” OR “patient”  “spoonies” AND “pain” |

The chart below depicts the varying types of media analyzed for this audit.

Key Themes

Several key themes emerged across all of the articles. Conversations focused on interactions between providers and patients as well as recently released clinical guidelines and federal and state recommendations from the National Institutes of Health’s National Pain Strategy and the Center for Disease Control’s “Guideline for Prescribing Opioids for Chronic Pain.”

Although the majority of the analyzed articles focused on chronic pain, with a combination of conditions, some specific conditions such as arthritis, osteoarthritis, and back pain in addition to others indicated in the chart below were mentioned with regard to long-term pain:

| **Conditions Mentioned** | |
| --- | --- |
| - Arthritis - Osteoarthritis - Back pain - Fibromyalgia - Sciatica - Headaches - Migraine - Neck pain - Knee pain - Joint pain - Spine injuries - Cancer pain - Compartment syndrome | - Stroke - Compression fracture - Erythromelalgia - Soft pain tissue syndromes - Pelvic pain - Obesity - Injury - Mental disorders - Mitochondrial diseases - Musculoskeletal pain - Deep internal pain - Postural orthostatic tachycardia syndrome - Sjogren’s rheumatologic problems |

The following modalities were mentioned within the analyzed articles:

| **Complementary Modalities** | |
| --- | --- |
| - Acupuncture - Yoga - Meditation - Mindfulness - Natural products - Herbal medicine - Cognitive-behavioral therapy | - Chiropractic or osteopathic manipulation - Massage therapy - Probiotics - Tai chi and qi-gong - Hypnosis - Chinese medicine and herbs - Relaxation - Biofeedback |
| **Other Modalities** | |
| - Physical therapy - Cannabis | - Reiki - Exercise |

Key Questions: Patients

**Q. What challenges do patients face when communicating about pain to their health care providers?**

**A: Stigma, lack of understanding, lack of compassion, risk of rejection, shortage of time.**

*“I think the most difficult part of having chronic pain is that most people don’t understand it. There’s a large difference between acute pain, which everyone has experienced, and chronic pain. Chronic pain is not just acute pain that lasts a long time. And because I look well much of the time, people don’t understand that I’m in pain. And if I’m not in pain at the moment, I could be any second. It can come on very suddenly.”*

**Beth Thorp**, chronic pain patient ([source](http://www.practicalpainmanagement.com/pain/living-managing-chronic-pain-patient-story))

Patients face a multitude of challenges when discussing their pain. They face stigma – not only from the public which may not understand the gravity of chronic pain, but from those who should be their champions: their health care providers. They run the risk of being turned away or “[rejected](http://www.painnewsnetwork.org/stories/2016/5/6/iowa-pain-patient-on-hunger-strike)” due to stricter prescribing guidelines and monitoring systems; many of which [lacked patient involvement](http://www.painnewsnetwork.org/stories/2016/3/15/d4tpwjywebx7t1yswltusmf86bmg31) in development. In addition, patient-provider interactions are often confined to a very short timeframe.

An anecdotal sample of media articles from 2015 identified by a search for “helping patients talk about pain”, when compared with articles using the same terms from January through August 2016, showed a shift away from [supportive](http://www.nytimes.com/2014/07/13/opinion/sunday/how-to-talk-about-pain.html) attitudes regarding patients toward more stiff and [skeptical](http://reason.com/blog/2016/03/16/cdc-prescription-guidelines-will-leave-m) [coverage](http://www.nytimes.com/2016/03/17/health/er-pain-pills-opioids-addiction-doctors.html) focused on dependency and inadequate pain management as responses to the opioid crisis took hold.

- ***Insight:*** Straightforward guidance for patients to talk to their providers about complementary and integrative therapies for pain is insufficient given emotional, psychological and logistical barriers
- ***Implication:*** Patient-focused campaign communication should take a pragmatic and empathetic tone, acknowledging the challenges of having a nuanced pain conversation with a provider and supplying tools and support for a collaborative relationship. For example, “insider” tips from a provider on how to approach a pain management interaction could be helpful for patients.

*“At a time when patients need to tell their story, need to feel that their pain is well understood before they can move forward, we are cutting them off at the knees,”*

**Dr. Joshua Wootton**, director of pain psychology at the Arnold Warfield Pain Center at Beth Israel Deaconess Medical Center ([source](http://boston.cbslocal.com/2016/01/14/chronic-pain-stories-of-struggle-and-hope-part-8-challenges/))

**Q. What kind of information influences patients’ decisions about self-care for pain?**

**A: Provider interactions, media coverage, other patients.**

*“I’m trying to get into a clinical trial for a new chronic-pain drug. You’d think I was competing for a seat on the Virgin Galactic space shuttle.”*

**Ellen Sue Stern,** chronic pain patient ([source](http://www.startribune.com/how-not-to-treat-chronic-pain-the-way-we-do/370333521/))

It is widely known that health care provider opinions play an important role in guiding patients’ decisions about treatment. These conversations can also serve as informative consultations to present new or lesser-known options available to integrate into a treatment plan. Though this is the ideal situation, there are additional factors at play before the conversation takes place in the clinical setting. Two major caveats for patients that appeared within this media audit were the fear of dependence on opioids and a hesitance to use them due to associated stigma. Articles exploring pain management options suggested patients ask their provider a few [key questions](http://www.cnn.com/2016/05/11/health/opioid-prescription-painkillers-patient-advice/) before accepting an opioid prescription, and perhaps most helpfully encouraged patients to find a collaborative partner in “what is likely to be a [long relationship](http://www.everydayhealth.com/columns/health-answers/beyond-opioids-other-options-treating-chronic-pain/)” – working with a physician to develop a treatment plan combining traditional medicine with complementary therapies for long-term pain management.

Though these suggestions were useful, those two major caveats still resounded in patient-penned anecdotes. Cost, insurance coverage and public or peer perception of complementary and integrative approaches were also deemed influential in the decision-making process and often led to patients seeking care outside of the clinical setting. A patient with rheumatoid arthritis [described](https://www.theguardian.com/us-news/commentisfree/2016/jun/22/chronic-pain-management-medication-opioids-prescription-drug-epidemic) the stigma she navigated, even though she integrated a number of nonpharmacologic approaches into more traditional care: "I spend a lot of time giving myself the support that the medical establishment cannot provide."

- ***Insight:*** The opioid epidemic has already changed the way patients feel about approaching providers for help with pain. A “them vs. us” sensibility may be emerging between patient and provider communities – and patients may turn to self-care for pain as a true “alternative.”
- ***Implication:*** While an NCCIH campaign will not be an antidote to the opioid epidemic, campaign messaging can be constructed with a view to disarming patients and providers who may feel mutually ill-equipped to engage on pain management. Evidence regarding the benefits of an integrated pain management approach, rather than simply the efficacy of certain specific treatments, should be identified and underscored.

**Q. What resources do patients use for information on complementary health approaches?**

**A: Lifestyle media, top-tier media, pain-based and lifestyle-based communities (online and offline)**

Patients used a variety of resources for this information outside of consultation, if any, with their providers. Lifestyle sites such as [Refinery29](http://www.refinery29.com/lower-back-pain#slide) included these approaches in list-style articles, often citing modality experts. A number of articles discussed community groups such as online support groups and those within specific communities, such as the veteran population. One advocate who established a pain-focused support group summed up the opportunities for connection online: “It’s free, easy to join. It’s been amazing to watch and listen as these groups have developed. People are being helped. There’s nothing quite like peer-to-peer support for chronic pain sufferers.”

These groups, like any social group, have great potential to expand information sharing about complementary health approaches within the scope of conversations such as “what worked for me” or “my doctor recommended this approach.” However, as health care increasingly moves away from a one-size-fits-all approach and knowing the individual experience of pain varies greatly, it is important to note that this information is not always used productively or accurately.

In general, patients tap into mass media outlets for information. The topics covered in the “Key Themes” section were often dominant; complementary health approaches appeared anecdotally or within reporting on research. A better question to serve this research process might have focused on what resources are *available* for patients rather than what they used, as the former was easier to garner from media coverage.

- ***Insight:*** As patients digest media reports about pain management and exchange them with others on social media, there is huge potential for popular information on complementary health approaches to spread and have impact – as well as a risk that scientific nuances, caveats and patient heterogeneity may be overlooked. Patients in pain look for hope, connection, and relief, which may influence their interpretation of data and the sources they most trust.
- ***Implication:*** NCCIH should weave subtle guidance on evaluating and applying scientific findings into campaign messaging, as well as working with popular pain influencers to guide dissemination of information in networks trusted by patients.

Key Questions: Providers

**Q. What are providers’ current unmet needs in pain management communication to patients?**

**A: Lack of time, burnout, lack of knowledge on safety and efficacy of nondrug approaches.**

Many articles mentioned the risk of physician burnout and limited time to explore alternative options to pharmacologic pain treatment with patients. Other unmet needs, explored in more detail via the following key questions, included a solid understanding about the efficacy of specific complementary and integrative approaches that can be used for chronic pain. Media coverage has not placed a huge emphasis on navigating the patient-provider relationship in regard to pain; rather, it focused on guidelines and their implications, often in light of the opioid epidemic.

- ***Insight:*** For providers, the nature of interactions with patients about pain management has become even more formal and loaded with potential pitfalls in the wake of guideline changes due to the opioid epidemic. Media coverage focused on drama and conflict may exacerbate perceptions of this shift.
- ***Implication:*** NCCIH should invest in development of guidance for providers on how to foster collaborative/consultative relationships with chronic pain patients. This may encourage more providers to initiate conversations about complementary and integrative approaches, since these modalities tend to require long-term practice before benefits are seen.

**Q. What resources are available to help providers communicate risks and benefits of pain management options to patients?**

**A: Medical training, clinical guidelines, federal and state recommendations / policies, professional associations, trade media, senior / leading professionals.**

*“When a prescription is necessary, we should try the safest and most effective medication first, and pain treatments that come with such risks should be the last option. Non-opioid therapies, physical therapy, cognitive behavioral therapy, and interventions like steroid injections, are alternatives that can lessen pain and improve function with far fewer risks than opioid analgesics,”*

**Dr. Tom Frieden,** Director, Centers for Disease Control and Prevention ([source](http://www.huffingtonpost.com/tom-frieden-md-mph/do-no-harm-cdc-guideline_b_9471168.html))

[Pain-focused training](http://www.huffingtonpost.com/tom-frieden-md-mph/do-no-harm-cdc-guideline_b_9471168.html) for health care providers was often seen as inadequate and many stakeholders on the patient and provider side called for collaboration to address too-brief or incorrect training procedures. Guidelines such as those most recently released by the CDC aimed to address resource gaps, but stakeholders [noted weaknesses](http://www.practicalpainmanagement.com/resources/news-and-research/responses-criticisms-over-new-cdc-opioid-prescribing-guidelines) in the document, such as its evidence base.

Studies on the efficacy of pharmacologic approaches show [weak evidence](http://www.upi.com/Health_News/2016/05/26/Study-Opioids-not-effective-for-long-term-back-pain/5451464269189/) for their use in long-term painful conditions. Lynn Webster, M.D., a past president of the American Academy of Pain Medicine, penned an [opinion piece](http://www.sltrib.com/opinion/4191773-155/op-ed-to-solve-opioid-crisis-we) suggesting patient‒provider relationships supplemented by a strong, up-to-date evidence base: "It is imperative that we allow compassion to lead us forward while science lights the way.”

*"Most of all, we should promise our patients we won’t abandon them. We may elect in certain circumstances to stop an opioid prescription, but seldom should it be done abruptly and never as the final chapter of care."*

**Dr. Tim Lahey,** Director of Education, Dartmouth Institute for Health Policy & Clinical Practice, Geisel School of Medicine ([source](https://www.statnews.com/2016/07/26/opioid-contracts-addiction-legislation/))

- ***Insight:*** It may be frustrating for providers that guidelines based on weak evidence are changing the standard of care – particularly since nonbinding guidelines are often translated into more binding regulations by providers’ local and state boards and health systems.
- ***Implication:*** Guidance for providers on integrating complementary and integrative approaches into patient pain management plans as part of a stepwise approach may be helpful. This might be even more resonant if it was informed by providers who have also been or are chronic pain patients themselves.

**Q. What resources do providers use for information on complementary health approaches?**

**A: Clinical guidelines, Federal and state documents, medical training, interactions with other providers, academic institutions**

[There](http://www.huffingtonpost.com/healthline-/treating-pain-in-the-mids_b_10153542.html) was an identified education gap in pain management strategies for clinical practice guidance that reflects the multifaceted nature of the pain experience and an increasing need for integrated treatments. Providers are equipped with prescribing guidelines, recommendations such as the National Pain Strategy and continuing medical education (CME) activities. From this audit, it appeared as though many of these resources simply mentioned nonpharmacologic approaches as alternatives rather than diving into an examination of their safety and efficacy.

- ***Insight:*** Existing Federal guidelines on pain management in light of the opioid epidemic may not be a sufficient resource base for providers who would like to learn more about how complementary and integrative health approaches could help their patients, particularly those who have been relying on opioids.
- ***Implication:*** NCCIH can leverage its authority as part of a Federal health agency to credibly educate providers on how to translate the spirit of the CDC guidelines into practice.

**Q. What challenges do providers face in navigating pain management decisions with patients?**

**A: Lack of trust, conflicting pressures of patient need and malpractice threats / professional censure, inadequate empathy, challenges of setting boundaries, reluctance to treat pain, scant evidence base, lack of consensus within the medical community and Federal health agencies.**

*“With chronic pain, you have to have that trust and relationship, to help people feel like people.”*

**Robert Wergin,** president of the American Academy of Family Physicians ([source](https://www.washingtonpost.com/national/health-science/how-a-yoga-injury-lead-me-into-the-crossfire-over-opoids/2016/02/01/ccae46a6-87f1-11e5-9a07-453018f9a0ec_story.html))

Physicians faced the challenge of building trust and incorporating best practices in prescribing in the real-time treatment setting. Other obstacles to effective pain management included discomfort treating chronic pain, inadequate empathy for pain complaints and failure to set appropriate boundaries for scheduled pain medications.

Many articles noted the ["narrowness" of evidence for CDC guidelines](http://www.practicalpainmanagement.com/resources/news-and-research/cdc-issues-final-guidelines-opioid-prescribing) as well as conflicting perspectives from NIH and other federal agencies. These challenges can lead to a lack of cohesive guidance for providers dealing with an already-difficult situation.

- ***Insight:*** With the alarm accompanying the opioid epidemic, providers may be losing perspective on how much their expertise and care can mean to patients. From the other side, patients may be hampered in seeing their providers as allies and expert resources.
- ***Implication:*** Campaign messaging could highlight the emotional support providers can offer to patients and the meaningful nature of their role in patients’ lives. Case studies may be useful.

**Q. What are common sentiments among providers around nonpharmacologic approaches for chronic pain?**

**A: Some stick to “tried-and-true” conventional care; some look for more options; many encourage self-care. Most general practitioners don’t have detailed knowledge of non-drug approaches. This may change as providers see approaches work effectively in practice, for example within the VA system.**

Some providers may not be inclined to move away from the treatments they’re accustomed to prescribing, and others [fully support](http://vtdigger.org/2016/03/27/james-mcdaniel-curbing-opioid-use-with-alternative-approaches-to-pain/) the early consideration of non-opioid pain relievers or nonpharmacologic options. As noted in the literature review, self-care was upheld as one of the best methods of treatment for chronic pain.

“The approach of the conventional medical community to things like acupuncture is really changing a lot… When I was in residency or medical school I did not receive training (on issues such as) when do you refer for acupuncture (or) when do you think of yoga. I’ve learned a lot recently; I was trained in acupuncture by the VA.”

**Dr. Julie Franklin,** White River Junction VA Medical Center ([source](http://www.concordmonitor.com/Articles/2016/03/From-Archives/vetsOpioid-cm-030516))

This media audit found that while most providers who shared commentary or penned articles regarding pain treatment were supportive of using nonpharmacologic approaches, they did not explicitly share information on implementation or preferred methods unless they were a specialist, such as a chiropractor.

- ***Insight:*** The sheer scale of patient need, as well as an expanding evidence base on safety and efficacy, may be driving more providers to get curious about complementary and integrative approaches.
- ***Implication:*** NCCIH could legitimately position its pain campaign as a response to increasing demand; this could be one way to avert promotional or heavy-handed overtones.

**Q. Which stakeholders are discussing chronic pain? Where are they having these discussions? AND who are the gatekeepers of information on chronic pain for both HCPs and patients/patients?**

**A: The following organizations appeared repeatedly in analyzed media coverage and/or are also important to NCCIH. If the latter was the primary criterion for inclusion, the organization appeared at least once in media coverage.**

| **Federal Stakeholders** | - Centers for Disease Control - Department of Veterans Affairs - National Center for Health Statistics - Center for Chronic Disease Outcomes Research |
| --- | --- |
| **Physician Organizations** | - American Academy of Pain Medicine - American Academy of Pain Management - American Pain Society - Physicians for Responsible Opioid Prescribing - American Medical Association - American Chiropractic Association - American Psychological Association |
| **Patient or Condition-Specific Organizations** | - Association of Chronic Pain Patients - National Fibromyalgia and Chronic Pain Association - International Pain Foundation - Mothering With Chronic Pain (blog) - American Chronic Pain Association - American Headache Society - American Migraine Foundation - National Headache Foundation - Live Support Group - ButYouDontLookSick.com - Fibromyalgia Inspirational - Suffering the Silence - Global Genes - Pain Free Patriots |
| **Academic Health Institutions** | - Institute of Medicine - American Association of Colleges of Nursing - Division of Pain Medicine, Stanford University - Wake Forest Baptist Medical Center - Center for Complementary and Integrative Medicine, Division of Rheumatology, Tufts Medical Center in Boston - Arnold Warfield Pain Center at Beth Israel Deaconess Medical Center |
| **Modality-Specific Organizations** | - North American Spine Foundation - Foundation for Chiropractic Progress - Massage Therapy Foundation - Evidence for Massage Therapy Working Group (led by the Samueli Institute) |
| **Industry Stakeholders** | - Travelers Early Severity Predictor (Travelers) - Quell |

- ***Insight:*** Though the academic, government, clinical, industry and patient communities may not come together around pain management in practice, in the media they are all impactful.
- ***Implication:*** NCCIH could bring focus groups together that include representatives from several of these influencer perspectives to clarify gaps and key messages for each group, as well as identify areas of shared purpose.

**Q. What are stakeholders saying about complementary and integrative approaches to chronic pain?**

**A: Verbally supportive while challenges like poor insurance coverage, low access, high costs and time commitment persist in practice.**

Stakeholders generally nodded to complementary and integrative approaches as supplementary options for patients with chronic pain, but said that lack of insurance coverage or [access](http://www.pbs.org/wgbh/frontline/article/veterans-face-greater-risks-amid-opioid-crisis/) may deter prescribing or use. Some coverage suggested that well-covered complementary and integrative approaches for chronic conditions may negatively impact work attendance. A [recent study](http://tcbmag.com/News/Recent-News/2016/August/Alternative-Medicine-May-Increase-Workplace-Absent) suggested that use of certain types of mind and body approaches as well as natural products may correlate with more days of missed work due to illness or injury.

“Non-pharmaceutical approaches to relieving chronic pain such as cognitive behavioral therapy, exercise therapy, acupuncture, biofeedback, and the like are helpful for some individuals. Sadly, many Americans can’t access or afford these treatments. Not all health insurance plans cover such interventions, and not all health care providers are willing to prescribe them to their patients.”

([source](https://www.statnews.com/2016/06/09/opioid-abuse-compassion/))

Industry stakeholders have different interests than patients and providers within the pain landscape, but some have taken steps to assist early intervention. For example, [Travelers](https://www.bostonglobe.com/metro/2016/07/30/can-chronic-pain-prevented/hu93w9Q8N5oVEVtdbvfsMI/story.html), an insurance company that covers worker’s compensation cases, developed an “Early Severity Predictor” integrating options that may help prevent long-term suffering from chronic pain conditions. It uses a statistical model that considers medications used, mental health, musculoskeletal health and the presence of other medical conditions to identify risk for pain, then can contact providers to suggest interventions. An expert acknowledged more research is needed to understand which patients are at risk for chronic pain and which interventions work best.

- ***Insight:*** The questions of “who’s going to pay?” and “how do I get access to / learn how to do these approaches?” could be real barriers to patients and providers considering complementary and integrative approaches.
- ***Implication:*** NCCIH cannot counsel on insurance coverage or promote certain providers/instructors of complementary and integrative health approaches, but campaign messaging could stress the self-directed, free of cost nature of certain practices and encourage stakeholders/TPOs to share recommendations on how to practice. Insurers could be included in TPO and influencer lists for events such as Twitter chats.

**Q. What information might be missing from the national conversation on chronic pain?**

**A: How to avoid chronic pain in the first place and self-manage it when it can’t be prevented.**

“We spend billions on advances in pharmaceuticals, devices, surgeries and other innovative treatments for chronic pain, yet we fail to deliver long-term relief, primarily due to the lack of educating patients in self-management strategies to reduce these risk factors and enhance protective factors.”

([source](http://www.startribune.com/health-care-and-addiction-x2009-x2009-instead-of-opioids-prevent-chronic-pain/375905321/))

Patients with chronic pain represent about [30 percent](https://www.veterans.senate.gov/imo/media/doc/VA%20Clancy%20Testimony%203.26.20151.pdf) of the adult population in the U.S., yet primary care physicians often have little training in managing these conditions. Many experts said guidelines and recommendations cannot alone solve complex public health programs – several sources encourage shifting the focus on chronic pain from treatment to prevention.

- ***Insight:*** A campaign focused on complementary and integrative approaches for chronic pain management could easily create the perception that these approaches are only for treatment.
- ***Implication:*** NCCIH can highlight the use of complementary modalities for wellness and preventative care, leveraging NHIS and other data.

Topline Takeaways

As referenced in the NCCIH Pain Campaign Formative Research Approach, the purpose of this media audit was to leverage recent media monitoring reports on opioid overuse and the recently issued Centers for Disease Control report, as well as conduct additional searches to identify relevant themes in top-tier consumer media outlets, patient blogs, HCP blogs, federal government health agency blogs, professional society newsletters and other relevant sources.

Results from this audit provide a glimpse at the experiences of patients and providers working to treat and communicate with each other about chronic pain, leading to a few key takeaways:

- Patients face potential stigma, risk rejection and face a lack of understanding and/or compassion
- Provider interactions, media coverage and the opinions and suggestions from peers influence patients’ decisions about self-care for pain
- Providers work within limited timeframes for consultation and lack knowledge on the safety and efficacy of nonpharmacologic approaches
- Patients mention using coverage from top-tier outlets, lifestyle media and pain- and lifestyle-focused communities both online and offline for information on complementary health approaches
- For providers, pain management decisions made hand-in-hand with patients are complicated by lack of trust, conflicting pressures, inadequate empathy, difficulty setting boundaries, hesitance to treat pain, a limited evidence base and a lack of consensus on treatment options within the medical community and federal agencies
- While many providers encourage self-care, some stick to conventional care – and most do not have a solid grasp on nonpharmacologic approaches
- Though stakeholders are generally verbally supportive of complementary and integrative approaches, challenges such as low insurance coverage and limited access persist in practice

Opportunities for Future Research

Two formative research questions to which answers remained unclear following the literature review, regarding resources patients and providers use for information on complementary health approaches, were further clarified upon review of recent media coverage. There are gaps in both knowledge and evidence bases for complementary and integrative approaches related to pain management, creating space for NCCIH to provide resources that equip involved parties for informed, evidence-based decision making.

**Primary research opportunity:** Further research is needed to examine the information pathways that clinicians find most credible when it comes to “new” evidence (for example, data on the safety and efficacy of complementary and integrative health approaches) and which are most likely to lead to changes in patient interactions, recommendations and clinical practice.

The voice of the chronic pain patient was prevalent in media coverage, in contrast to the literature review. Whether through a patient community blog or an anecdote within a top-tier outlet’s article, firsthand experiences are powerful testaments to what’s missing from the national conversation on chronic pain treatment: productive discussions of how to prevent painful conditions in the first place, how the public and providers can show respect for pain patients’ experiences and how to safely self-manage when they cannot be prevented.

**Primary research opportunity:** Further research is needed to identify ways that chronic pain patients’ perspectives and suggestions can be incorporated into an NCCIH campaign and, potentially, into broader pain professional community dialogues about clinical best practice.

Conclusion and Next Steps

NCCIH is positioned to further the narrative of this public health issue by shedding light on information on nonpharmacologic approaches sourced from rigorous research, leveraging engaging, evidence-based information paired with strong patient and provider anecdotes. This must be done with sensitivity to the many constraints currently affecting pain management communication between patients and providers, which have been explored both within this document and the preceding literature review.

***Primary research opportunity*** As consultation for designing materials that help facilitate conversations about complementary health approaches between patients and providers, focus groups that bring the two communities together (providers and chronic pain patient advocates) may help elucidate: a) information pathways with mutual influence and b) verbal cues that may support the development of shared language for patient‒provider clinical interactions.

The next step of the formative research process for this campaign is an Audience Tracker report, which will leverage JPA’s proprietary tool to help further identify themes and stakeholders stemming from “issue bundles” of terms relevant to pain management, chronic pain, nonpharmacologic approaches to pain, and complementary and integrative pain interventions. The Audience Tracker report will also include themes from coverage of NCCIH’s Mayo Clinic Proceedings paper, *Evidence-Based Evaluation of Complementary Health Approaches for Pain Management in the United States*.
