# Supplementary material for: What Twitter teaches us about patient-provider communication on pain
Source: PLoS One. 2019 Dec 26;14(12):e0226321. doi: 10.1371/journal.pone.0226321 (PMC6932781; doi:10.1371/journal.pone.0226321)
Supplement: S4 File — (DOCX) [file pone.0226321.s004.docx]

**S4 File**

**Glossary**

1. **Social Network Analysis (SNA):** In this study, the term social network analysis (SNA) refers to a large network visualization of relationships between individual accounts on Twitter. The SNA maps in this study are composed of individual Twitter accounts, sometimes referred to as “nodes,” which are connected to one or more accounts in the map via social relationships. The SNA maps allow for insight into both social structure and flow of information.
2. **Nodes:** Individual Twitter accounts within the SNA maps.
3. **Engagement:** Engagement refers to follow connections between individual accounts (or nodes) within the SNA maps, or details in the information sources that these accounts are citing within their Twitter posts.
4. **Relationships:** Similar to “engagement,” relationships refer to follow connections between individual accounts (or nodes) within the SNA maps.
5. **Interactions:** Similar to “engagement,” relationships refer to follow connections between individual accounts (or nodes) within the SNA maps.
6. **Seed Lists:** An initial list of accounts used to generate the SNA maps. The seed lists for the Pain and Oncology SNA maps within the study were comprised of accounts that were focused on posting on Twitter about pain and oncology topics, respectively. The seed lists were then run through a clustering algorithm (hierarchical agglomerative clustering) to generate clusters of association within each map.
7. **Attentive Clustering:** A method of generating an SNA map that focuses on identifying clusters of association between nodes within the map. An initial seed list for each map was run through an algorithm (hierarchical agglomerative clustering) based on Twitter behavior to generate clusters of association. Subsequent to clustering, the map-making process then uses supervised machine learning to generate labels for clusters and groups (sets of related clusters) from a set of human-labeled examples. The machine-generated labels are then manually verified and modified by human coders, as needed, to reflect the account types (such as accounts owned by physicians) within each cluster.
8. **Density:** The proportion of connections between nodes in a network out of total possible connections. The follow relationships between audiences were compared by collecting the total follows between groups, and then applying a density measure to correct for disparate audience sizes.
9. **Sources:** Information sources (web domain URLs) cited by accounts within the SNA map within their posts on Twitter.
